# Active Plasmonic Surfaces via Electrically Driven Actuation of DNA-Tethered Nanoparticles

**DOI:** 10.1021/acsnano.6c04919

**Published:** 2026-06-09

**Authors:** Mohammed M. A. Al Hussain, Abraham Kipnis, Anna Lumppio, Narat Witwiyaruj, Sesha Manuguri, Xuan-Hung Pham, Pierre Bléteau, Maxime Fauconnier, Mohammadmahdi Asgari, Viktar Asadchy, Anton Kuzyk, Kosti Tapio

**Affiliations:** † Department of Neuroscience and Biomedical Engineering, 174277 Aalto University, Espoo 02150, Finland; ‡ Department of Electronics and Nanoengineering, Aalto University, Espoo 02150, Finland

**Keywords:** active plasmonics, DNA, nanoparticle-on-mirror, nanoparticles, self-assembly, electromechanical actuation

## Abstract

Nanoparticle-on-mirror (NPoM) plasmonic surfaces (PSs) exhibit a rich ensemble of interesting optical properties, including strong field enhancement and vivid structural colors. NPoMs can be easily fabricated via the drop-casting method, and their optical responses can be tailored by, for example, the size, morphology, and material composition of the nanoparticles and/or the thickness of the spacer layer between the nanoparticles and the metal film. Despite the ease of fabrication, implementing active modulation of optical responses in NPoM PSs has remained challenging. Here, we demonstrate the realization of electrically driven NPoM active plasmonic surfaces (eNPoM). Electric potentials are used to modulate the distance between the DNA-tethered metal nanoparticles and the metal film, which leads to a strong change in the optical response. Our eNPoM displays large reflectance modulation in the visible spectral range at frequencies beyond 1 kHz. Moreover, our fabrication process can be combined with standard lithography methods to arrange nanoparticles at predefined locations while retaining functionality. These results provide an approach to lithography-complementary fabrication of active plasmonic surfaces with strong and reversible modulation of optical responses.

## Graphical abstract

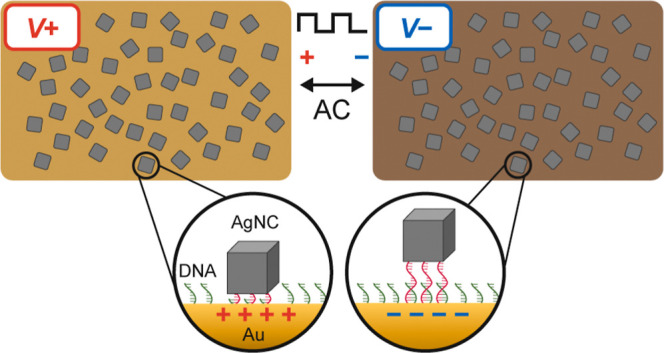

Plasmonic surfaces with dynamic and programmable light responses hold promise for a broad range of applications in computing, sensing, and display technologies.^1–4^ The dynamic behavior in these systems is typically implemented by applying external stimuli (e.g., electromagnetic,^5–7^ chemical,^2^ mechanical,^8,9^ thermal^10^) which change either material properties (e.g., refractive index)^6,11^ or geometry.^12,13^ While material-based approaches^2,6^ are widely adapted, particularly for metasurfaces,^7,12,14,15^ the geometry-based approaches^5,16–19^ have been utilized for sensing applications.^20,21^

Localized surface plasmon resonances (LSPR) of colloidal metal nanoparticles (MNPs) provide a versatile toolbox for both the manipulation and characterization of the optical responses in nanoscale systems.^22,23^ Furthermore, coupling of MNPs to a metal film enables the realization of the so-called nanoparticle-on-mirror (NPoM) plasmonic systems^24^ with optical responses tunable across a broad spectral range. In particular, NPoM-based plasmonic surfaces have been actively investigated for their utility in plasmonic color generation^25–27^ and extreme field enhancement.^28–30^ Optical properties of NPoM are, however, typically fixed after fabrication, which greatly limits their applicability. While several strategies based on refractive index change were proposed for active modulation of optical responses in NPoM surfaces,^31–34^ the spatial reconfiguration route remains largely unexplored.

Here, we report electrically controlled dynamic nanoparticle-on-mirror (eNPoM) plasmonic surfaces that utilize programmable hybridization and the mechanical flexibility of DNA and electric potentials for modulation of spatial configurations and, hence, optical responses. In our approach, the distance and coupling between the metal nanoparticles (MNPs) and the metal film are modulated by the voltage applied to the film. Modulation of the distance, in turn, changes the resonance absorption of the gap plasmon mode, which is observed in the far field as a change in reflectance in the visible spectral range. Our eNPoM operates at low voltages and exhibits a 211% relative change in reflectivity at 550 nm. Furthermore, eNPoM displays high actuation reversibility up to the kHz frequency range. Importantly, eNPoM fabrication can be combined with lithography methods to arrange MNPs into well-defined patterns and arrays.

## Results and Discussion

### Operation Principle of eNPoM

The eNPoM plasmonic surfaces were generated by tethering silver nanocubes (AgNCs) to a gold film via DNA, as shown in Figure 1a. AgNCs were chosen for their low-loss electric-field confinement and strong optical resonances in the visible spectral range.^35–37^ The AgNCs were coated with two different single-stranded DNAs (ssDNA), with the short passivating ssDNA (blue) setting the density of the longer strand (red) acting as an anchor (Figure 1a). The gold surface was functionalized with 6-mercapto-1-hexanol (MCH) and substrate ssDNA (green) complementary to 6 nt of the anchor strand. Using shorter molecules (MCH at the gold film and passivating DNA strands at AgNCs) lowers the material density between the particle and the surface, allowing for smaller particle-film gap distances during the electrical actuation of AgNCs. The anchor strand is deliberately diluted by a 90:1 excess of passivating strands, resulting in approximately 8 anchoring strands per cube face (Supporting Information Section 9). This sparse anchoring provides the particle with mechanical freedom to move under the applied electric field while maintaining stable tethering via multivalent DNA hybridization. Details of AgNCs and gold film functionalization are provided in the Methods section and Supporting Information.

**Figure 1:**
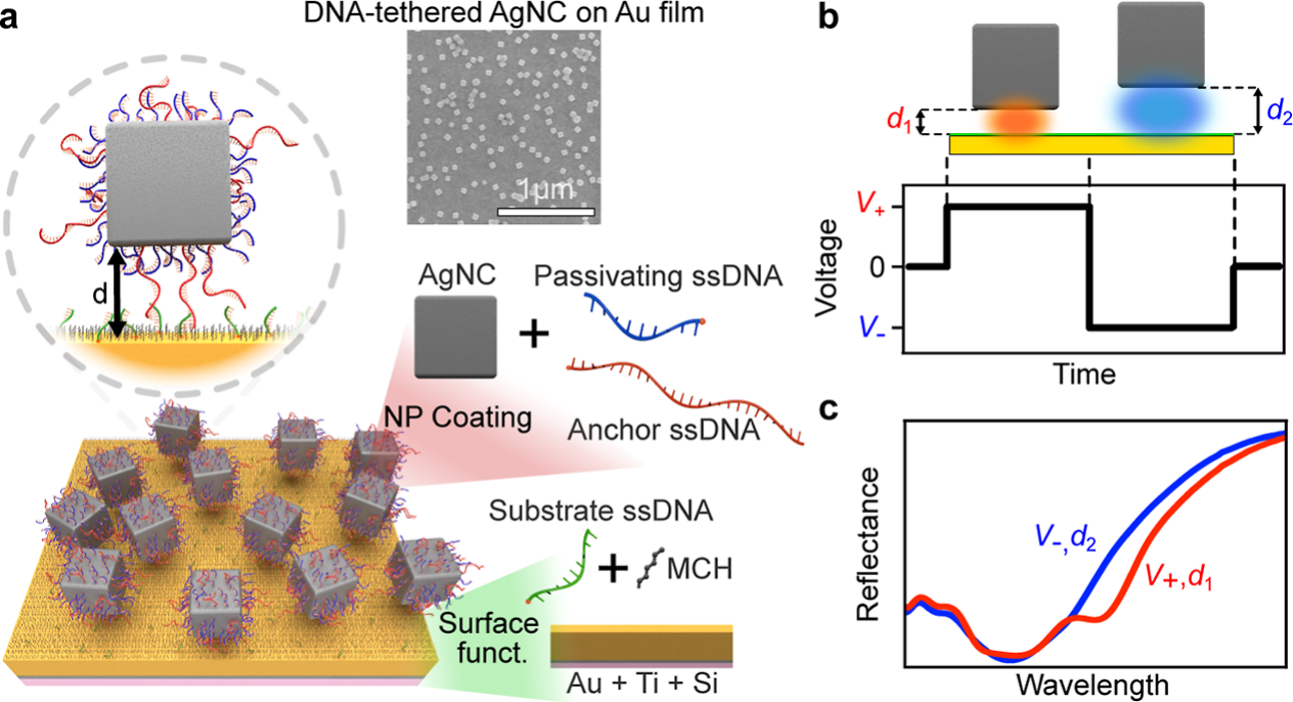
Operation principle of the electrically driven particle-on-mirror (eNPoM) system. (a) Schematic illustration of eNPoM. The layered structure consists of Au and Ti on a silicon substrate and is functionalized with thiolated ssDNA strands (substrate strand) and 6-mercapto-1-hexanol (MCH). Silver nanocubes (AgNCs) are coated with two different ssDNA strands, with the anchor strand containing a segment complementary to the substrate strand. After coating and functionalization, the AgNCs are drop-cast to form DNA-tethered AgNCs on the gold film, where the particle-to-surface distance d can be controlled by an electric potential. (b) Applying a positive (negative) electric potential to the gold film results in pulling (pushing) AgNCs toward (away from) the surface. (c) The active modulation of plasmonic responses is characterized by measuring the reflectance of the eNPoM film. Modulation of the distance between the AgNCs and the gold film results in a shift of the distance-dependent gap plasmon resonance and a change in the reflection spectra.

The dynamic actuation of AgNCs is realized by applying an electric potential difference between an indium-tin-oxide (ITO) counter electrode and the gold film (see Supporting Information Section 11 for more details). Electrostatic interaction between negatively charged DNA and the electric potential applied to the metal film exerts a force on the DNA-coated AgNCs, which are then either repelled or attracted to the surface. Changes in the AgNC-substrate gap distance alter plasmonic coupling between the AgNCs and the surface, leading to a modulation of the eNPoM surface reflectance (Figure 1c). To evaluate the performance of our eNPoM, we characterize the ensemble reflectivity of gold films with 8–20% AgNC coverage under constant-voltage biasing and switching performance (i.e., rate, reversibility, modulation) using an AC square-wave voltage.

### eNPoM under DC Voltage Bias

Our eNPoM operates in an aqueous buffer at room temperature. Several factors influence the optical responses and switching behavior: (i) the ssDNAs act as flexible anchors for the AgNCs, (ii) the particles can be moved by an applied electric field, where the anchor strand length and elastic properties set the nanoparticle range of motion, and (iii) the buffer conditions (i.e., ionic strength and conductivity) affect the screening of electrostatic interactions. To keep AgNCs tethered to the surface via DNA hybridization, we immersed the samples in *Tris*-ethylenediaminetetraacetic acid (*Tris*–EDTA-Na_2_ or TE) buffer containing sodium chloride (NaCl). The characteristic effect of buffer conditions on optical responses of eNPoM is presented in Figure 2, where we applied a constant bias between the gold film and ITO and measured the reflectance of the eNPoM with 26 nt anchor strand in 0.5 × TE-buffer supplemented with 0, 10, or 100 mM NaCl (for eNPoMs with 12 nt and 20 nt anchor strand lengths, see Figures S1 and S2).

**Figure 2:**
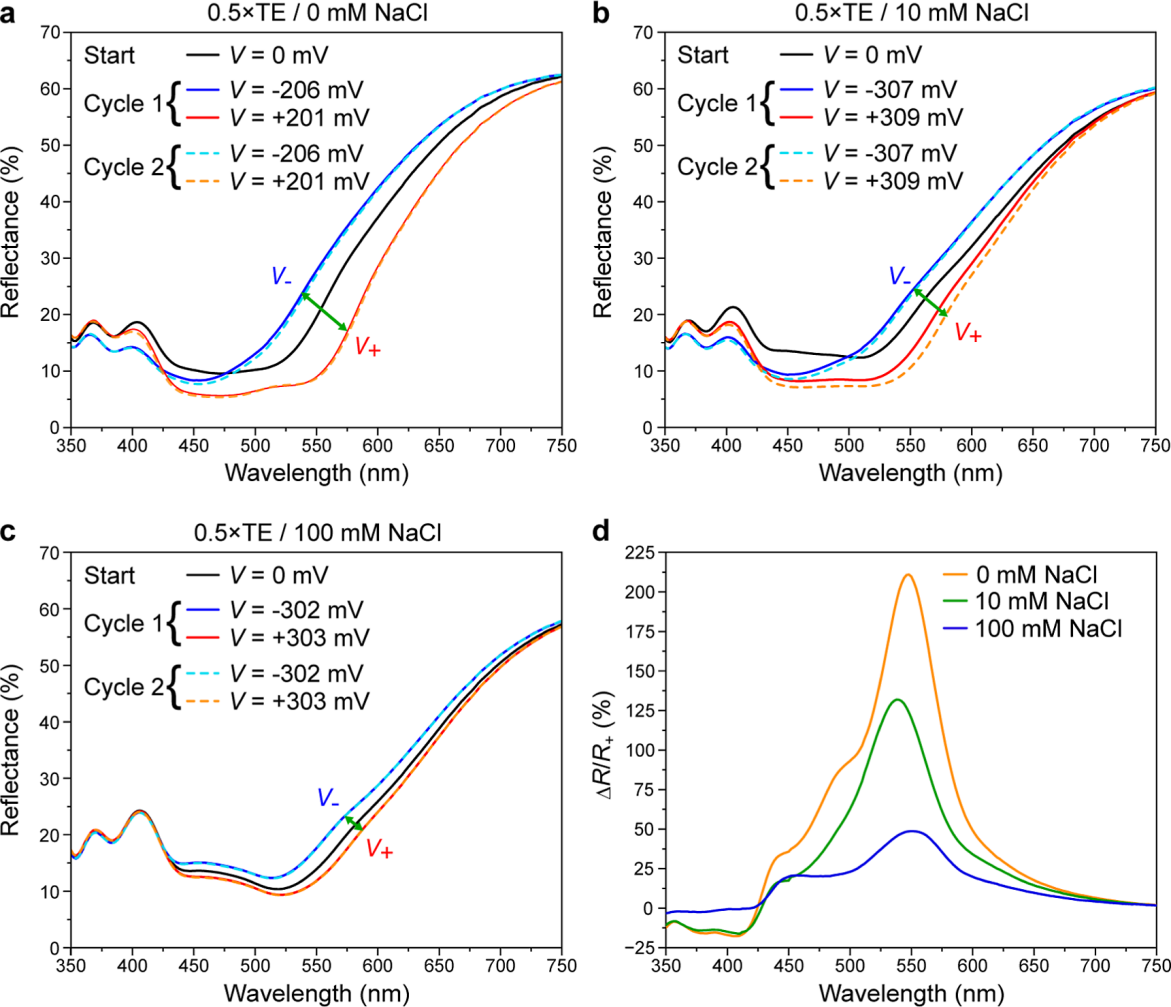
Reflectance measurement of eNPoM films and effects of ion concentration. (a–c) The reflectance curves of the eNPoM in different buffers with different NaCl concentrations and voltages cycled twice between positive and negative values. The anchor strand length is 26 nt. (d) The relative change in reflectance Δ*R*/*R*_+_ in different buffer conditions calculated from the curves in a–c.

We started each measurement with a baseline reflectance measurement at zero applied voltage, then measured at nominal bias voltages, resulting in a high but still reversible change in reflectance. The changes in reflectance were detectable around ±50–100 mV, and switching was noticeable, saturated, and reversible between ±200 and 400 mV depending on the NaCl concentration, which is comparable to other reported low-voltage actuators^15,38^ (see Supporting Information Section 5 for representative data).

The overall changes in reflectance at different applied voltages decreased as the NaCl concentration increased from 0 mM to 100 mM. We attribute this behavior to the increase in screening of electrostatic interactions between the film and DNA-coated AgNCs as the ion concentration rises. The baseline (*V*_in_ = 0 V) reflectance at different ion conditions is shown in Figure S3. A slight difference in the baseline reflectance at different NaCl concentrations could originate from the dependence of DNA persistence length on ion concentration.^39^ Importantly, the relative change in reflectance Δ*R*/*R*_+_ between negative and positive voltages is over 200% at ∼550 nm for eNPoM with anchor strands of 26 nt (Figure 2d). For the eNPoMs with shorter anchor strands of 12 nt and 20 nt, we observed comparable absolute modulation of reflectance in the range of 6.8–15.1% and 6.1–14.4% across the three buffer conditions, respectively. However, the relative change in reflectance was smaller, up to 32% and 134% (at ∼590 nm) for 12 nt and 20 nt, respectively (Figures S1 and S2). The eNPoM with 26 nt anchor strand displayed a broad shift in the reflectance without any discernible peaks. We attribute this to the size distribution of AgNCs and the flexibility of the DNA strand, which enables the particles to adopt different orientations and particle-to-surface distances. Conversely, when we used shorter anchor strand eNPoMs (Figures S1 and S2), the apparent dips and peaks in spectra become observable, indicating that the particle-to-surface distance *d* and possibly the orientation of AgNC are more uniform.

To further highlight the versatility of our method for the generation of active plasmonic surfaces, we also used gold nanocubes (AuNCs) to fabricate eNPoMs, as shown in Figure S4. AuNC-eNPoMs exhibited a smaller relative change in reflectance than AgNC-eNPoMs. The spectral differences between AuNC-eNPoM and AgNC-eNPoM extend well beyond the Au interband absorption near 500 nm. The dielectric functions of Ag and Au differ across the full visible range. Ag has a longer Drude relaxation time^40^ compared to Au,^41^ and its interband onset (∼320 nm)^42^ lies outside the plasmonic range, producing sharper and stronger resonances than Au. Furthermore, the gap plasmon resonance wavelength in NPoM systems depends on the nanoparticle material, not just the gap geometry, as previously demonstrated for Au, Ag, and mixed-metal NPoM constructs.^43,44^ Finally, the measured surface coverage differs between two particle types (AgNC fill fraction 18.1 ± 1.4% vs AuNC fill fraction 11 ± 1.8%, see Supporting Information), which directly affects the absorption depth of the ensemble reflectance.^24,37^ For applications requiring long-term stability, protective strategies such as the use of chemically inert gold-based NPs,^45^ AuNCs (Figure S4), offer viable routes to improved durability.

To obtain further insights into optical responses of eNPoM, we used Ansys High-Frequency Simulation Software (HFSS) for numerical simulations of reflectance at normal incidence and compared the results with measured spectra (see Figure S6). We calculated reflectance curves for a set of particle-to-surface distances (*d*) and computed a weighted average of the simulated reflectance curves over the cube size distribution to account for inhomogeneous broadening (Figure S5). The numerically simulated reflection spectra (Figure 3a) show redshifting of the peaks and dips as *d* decreases from 25 to 5 nm, characteristic of NPoM systems with thin-film spacers.^24^

**Figure 3:**
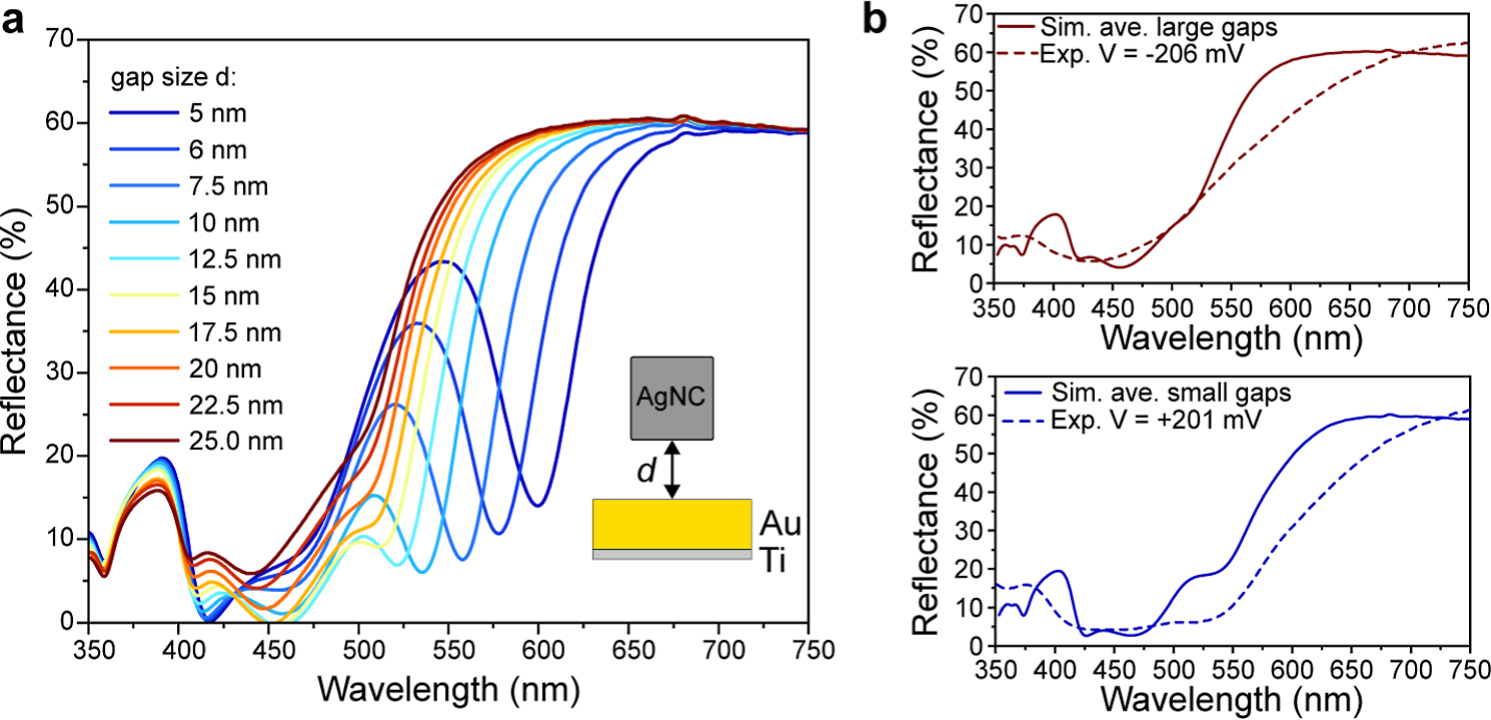
Comparison between the simulated reflectance of eNPoM and the experimental reflectance of ensemble eNPoM films. (a) The simulated reflection spectra of our eNPoM. Each curve represents the average spectra for a specific gap, accounting for the size distribution of the AgNCs (Figure S5). (b) The red and blue solid curves are constructed simulated spectra, where reflectance spectra from Figure 3a have been summed together with appropriate fitting factors. The dashed lines are the experimental curves from Figure 2a with 0 mM NaCl.

The gap medium was modeled as water (*n* = 1.33). We note that the presence of DNA in the gap raises the local refractive index (*n* ≈ 1.46 for ssDNA monolayers on gold).^46^ Simulations using *n* = 1.46 show a consistent red shift across all gap distances (Figure S6c), indicating that the simulation-extracted gap values depend on the assumed gap refractive index. Since the true effective refractive index lies between that of water and pure DNA, we use *n* = 1.33 throughout and note that the extracted gap distances should be interpreted as qualitative estimates rather than precise measurements. While we cannot decouple the effect of refractive index change and the particle-to-surface change, the former is affected by the latter due to compression of the DNA layer to a smaller volume when the distance is decreased, and our current hypothesis is that the main contributor to the reflectance shift is the gap modulation.

Evidently, no individual cube-size-averaged spectrum fully matches the experimental curves in Figure 2a. Still, the simulated reflectance above a 500 nm wavelength decreases with smaller gap sizes, which corresponds with our experimental data at positive bias voltages. To account for the film-particle gap heterogeneity inherent in tethered, particle-on-leash systems, for the reflectance curves with positive and negative DC bias voltages, we fit simulated spectra from Figure 3a to the positive and negative curves in Figures 2a, S1 and S2 using a least-squares fitting (see Supporting Information for more details). For the 26 nt anchor strand, the composite small- and large-gap spectra qualitatively agree with the experimental data (Figure 3b), suggesting that the average AgNC-to-film distance increases when the electric potential is switched from positive to negative.

Numerical simulations for eNPoMs with 12 nt and 20 nt anchor strands are shown in Figure S7, where similar behavior is observed as in the case of the 26 nt anchor strand. Comparing across all three strand lengths, the fitting suggested that the short 12 nt anchor strand functionalized AgNCs are closer to the film at positive bias than the other strands, consistent with longer tethers preventing the AgNCs from collapsing as close to the surface. In contrast, the fitting at negative bias is similar for all three strand lengths. This reflects the limited sensitivity of spectral matching at large gaps: as the gap increases, the gap-plasmon coupling weakens, and the reflectance features become shallower and broader, so that changes in gap distance produce negligible spectral differences.

The DNA tether from the gold surface to the AgNC consists of a 3 nt ssDNA spacer from the substrate strand (∼1.9 nm), a 6 bp hybridized dsDNA region (∼2 nm), and the free portion of the anchor strand, giving maximum tether contour lengths of approximately 8, 13, and 17 nm for the 12, 20, and 26 nt anchors, respectively (using 0.63 nm/nt for ssDNA^47^ and 0.34 nm/bp for dsDNA). The smaller gaps in our system arise from operation in an aqueous buffer, where the PVP coating on the AgNC,^44^ the passivating ssDNA, and the MCH layer collectively set a minimum gap of ∼5 nm. This operating range, from 5 to 17 nm, is in agreement with our simulations and supports our interpretation that the wide-range mechanical actuation of AgNCs produces the strong reflectance modulation reported here.

### Dynamic Switching Behavior under Alternating Voltage

To characterize the eNPoM film switching speed, we applied an alternating square wave voltage between the gold and the ITO and measured the optical response using a silicon amplified photodetector. A 300 mV amplitude square wave applied to the gold (Figure 4a) resulted in a corresponding modulated intensity signal of light reflected from the film (Figure 4b). We measured the amplitude roll-off *A*(*f*) as we increased the switching frequency for fixed voltage amplitude and fit the amplitude roll-off using an overdamped driven oscillator model $A\left(f\right)=A_{0}/\sqrt{1+{\left(f/f_{c}\right)}^{2}}$ (Figures 4d and S8). These results are consistent with previous works on switchable DNA layers, where actuation is tracked using fluorescence quenching by a metal film.^39^

**Figure 4:**
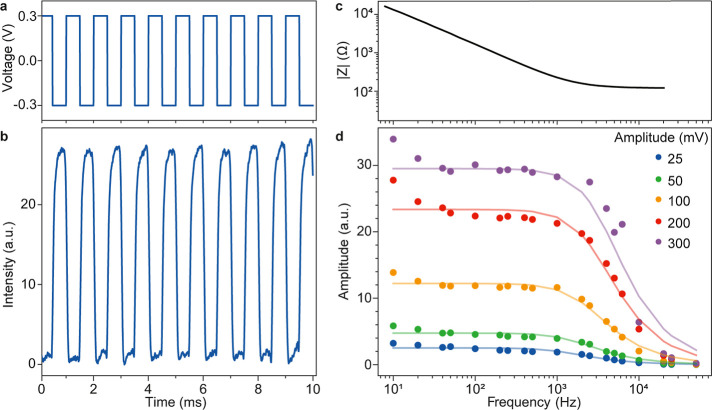
Ensemble eNPoM switching dynamics. (a) A square wave voltage with 300 mV amplitude is applied to the gold electrode. (b) Normalized optical response of eNPoM film (26 nt anchor in 0 mM NaCl 0.5 × TE buffer) in response to 300 mV square wave amplitude. (c) Electrochemical impedance measurement of an encapsulated liquid cell. (d) Reflectance modulation amplitude response as a function of driving frequency and applied voltage. The data fit an overdamped oscillator model (solid lines).

We approximate the switching energy of each eNPoM by measuring the current during cell charging and dividing the total energy by the approximate number of eNPoMs in the cell. We measured 200 μA peak charging current with a time constant of 3 ms to charge the cell from 0 V to 300 mV. Using AgNC surface coverage measured from SEM and cell surface area, we approximate 0.18 μJ charging energy of the cell and 0.6 fJ switching energy per cube (6.4 fJ per μm^2^). We also approximate the switching energy of each eNPoM by accounting for the electrostatic interactions between the electrode and DNA within the solvent electrostatic Debye layer (see Supporting Information for details).^48^ The electrostatic potential energy difference between positive and negative states in the case of the 10 mM monovalent ionic buffer is less than 0.02 fJ per eNPoM. The measured switching energy exceeds the calculated switching energy per eNPoM because not all energy input to charge the cell is converted into electromechanical actuation; efficiency losses arise from buffer heating and electrostatic double-layer charging in regions without cubes. Tethered AgNCs remained stable and switchable in encapsulated samples over the course of several days.

Additional data from high-speed camera measurements of the sample in a different electrochemical cell are presented in the Supporting Information (Figures S9–S13, Videos S1,S2,S3 and S4). The measured frequency response of switching is highly dependent on the liquid cell geometry. Parasitic capacitances and solution resistance in the liquid cell used for high-speed camera measurements reduce the maximum achievable switching speed to below 1 kHz.

### Controlled Switching of a Patterned eNPoM

Next, we assess the application of our technique toward multiplexed active plasmonic substrates. To demonstrate this, we patterned a gold film to form two isolated electrodes (the Aalto University logo letters and background) separated by insulating gaps, with ITO as a common ground. Both electrodes were driven out of phase (180° phase shift) using the same square voltage as previously. We recorded 1 Hz switching using a color camera under white-light illumination (Figure 5a, S14, and Video S5) and 1 and 1000 Hz switching using a monochrome high-speed camera under green LED illumination (Figures 5b and S15). Color camera data in Figures 5a and S14 show uniform color changes across the different surface parts, with a half-cycle square-wave voltage difference between the two video frames and a sensitivity of 4.4% normalized intensity change per mV at 0 mM NaCl, as shown in Figure S16.

**Figure 5:**
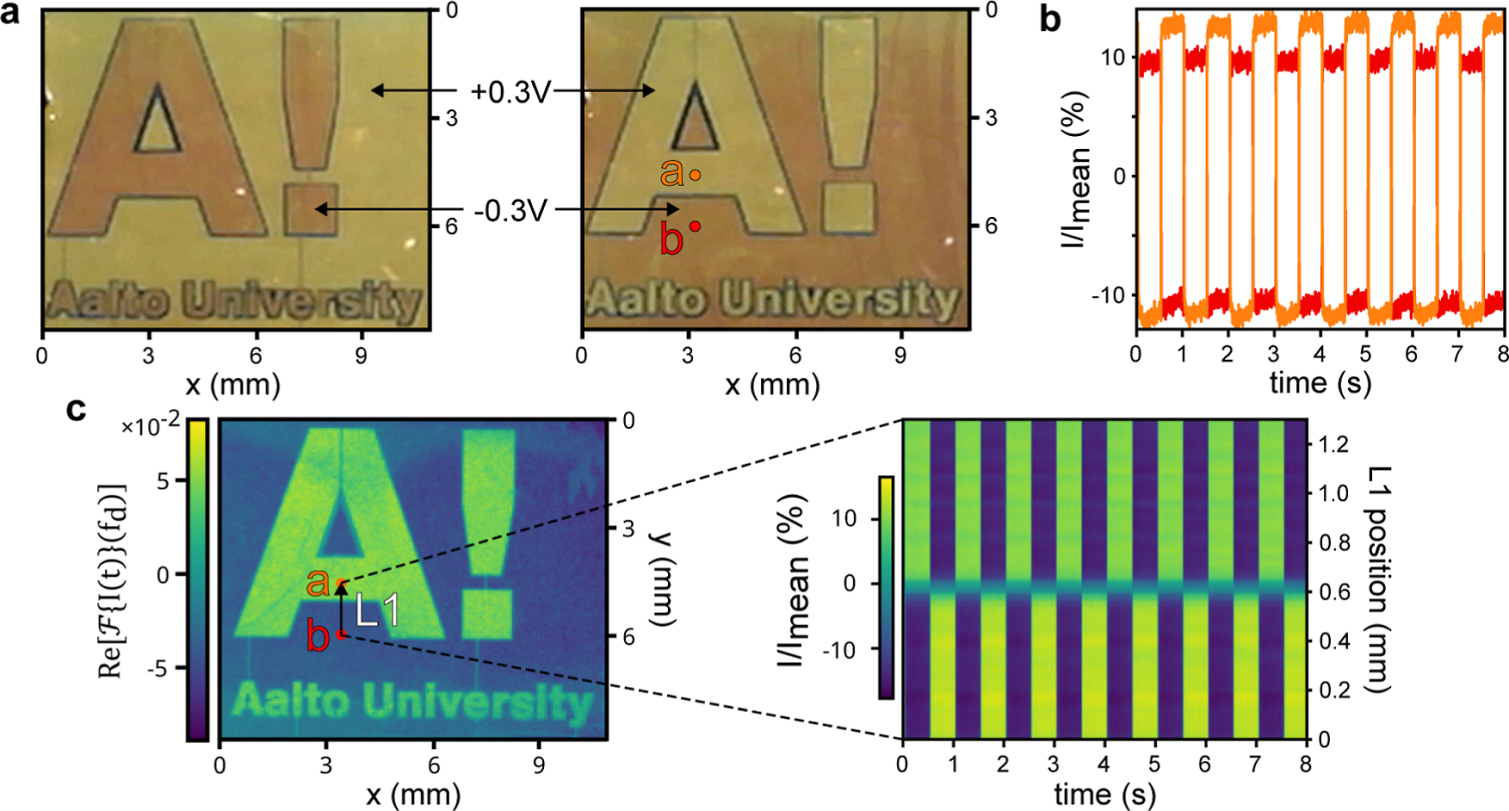
Cycling of the multioutput “Aalto Logo” sample. (a) Color images of the Aalto University logo patterned gold film, where the letters and background are controlled with two separate inputs and switched with a 180° phase shift. The left and right images have a half-cycle difference between them. The red and orange dots in the right image correspond to curves in b. (b) Normalized high-speed camera intensity data from the letter (red) and background (orange) corresponding to 10 × 10 pixel areas at the red and orange dots from a and c. Each curve is normalized by the mean value of the data set from the area. (c) The left image shows the real part of the complex Fourier transform map of the intensity, evaluated at the driving frequency (1 Hz). The right image shows the normalized intensity as a function of time over the line L1. The data was measured using a 1 Hz switching frequency, 0.5 × TE 0 mM NaCl buffer, 26 nt anchor strand length. Aalto logo patterned eNPoM. Credit: Aalto University. Logo used with permission.

Figure 5b shows the intensity values from the high-speed camera data at foreground and background sample locations (the red and orange dots in Figure 5a,c) while the sample was switched as described above. The observed intensity fluctuations agree well with our reflectance measurements (i.e., 20% change) and show the expected 180° phase shift between the two outputs at 1 kHz (see Figure S15). We also observed a clear phase difference in the intensity change of the background between points near and far from the 20 μm wide insulating gap between the two electrodes at 1 kHz and 0 mM NaCl (Figure S15). Ideally, each point on an electrode (background or logo) should have the same phase. Still, in-plane fields across the insulating gap may cause a crosstalk between the foreground and the background, which decreases with increasing salt concentration (Figure S15c). The crosstalk is detectable only in our high-speed camera data at a 1 kHz drive frequency and at NaCl concentrations below 100 mM, as shown in Figure S15.

To investigate areal switching uniformity, we calculate the Fourier transform of intensity at each pixel in the high-speed camera video, evaluated at the driving frequency (1 Hz), and show the corresponding color maps of the real part of the Fourier component in Figure 5c. These data indicate that our multi-input eNPoM exhibits near-uniform intensity changes between cycles, and all areas switch. Inhomogeneities may be due to differences in cube density,^37^ which could be reduced by more controlled sample preparation.^49^ Finally, we assessed the stability of eNPoM by continuous switching for 32 min at a 1 Hz drive frequency while recording reflectance every 10 min (Figure S17). The signal showed only slight degradation (*I*/*I*_mean_ decreased from 15.9% to 13.4%) during the measurement over 32 min (1800 cycles).

We also show that our eNPoM assembly can be combined with micro- and nanofabrication methods like EBL by patterning AgNCs into arrays with periods ranging from 300 to 600 nm. The patterning process is illustrated in Figure 6a, and it is based on the methodology previously used by Mirkin and Mulvaney groups.^50–52^ The basic principle is that a mask from poly­(methyl methacrylate) (PMMA) limits the surface functionalization by DNA and MCH and subsequent binding of AgNCs to the surface. Figures S18 and S19 show filling of a 600 nm period hole array with AgNCs and the corresponding fill fractions for empty holes or holes with either monomers or dimers. After removal of the PMMA mask, the arrays were reimmersed in the buffer, the actuation was characterized (Figure 6b and Video S6), and samples were then dried for dark field and SEM imaging (Figures 6c,d, S20 and S21). Figures S20 and S21 show two different patterned samples consisting of 40 μm × 40 μm arrays with periods ranging from 300 to 650 nm, after PMMA removal and in dry conditions. The 450 nm period array from Figure S21 is also shown in Figure 6c. Video S6 demonstrates reversible actuation of the arrays in Figure S20 under a dark field microscope in buffer, and Figure 6b shows the corresponding time-dependent actuation. Typical patterning results with corresponding yields are shown in Figures S22 and S23, with roughly 70–80% single particle occupancy with 3–11% of holes remaining empty. Increasing the time or particle concentration during the drop-casting typically results in full occupancy but also increases dimer and aggregate counts. We calculate the uniformity of electrically induced switching over the lattices shown in Video S6 and show the results in Figure S24, which indicate that approximately 10% of the lattice sites do not switch during voltage cycling. The reduced switching uniformity compared to nonpatterned surfaces indicates that the PMMA template confinement process lowers overall switching efficiency. Further improvements could be achieved by optimizing the hole size for more monodisperse AgNCs solutions, thus enhancing the blocking effect of AgNCs inside holes.

**Figure 6:**
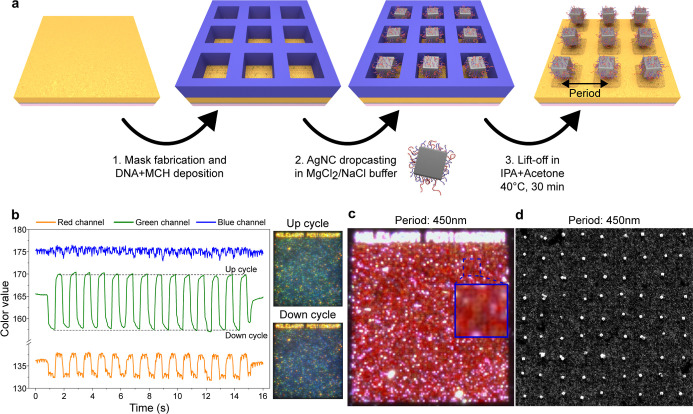
Active arrays of AgNCs. (a) Schematic view of the patterning process. A poly­(methyl methacrylate) (PMMA) mask is fabricated on top of a gold thin film using EBL, nanoparticles are drop-casted, and PMMA is removed to form the final patterned surface. (b) The time series data of an array with a 450 nm period actuated using a 200 mV AC square wave voltage. The images on top and bottom right show examples of the array during up and down cycles, respectively. (c) An example dark field image of a patterned AgNCs 40 μm × 40 μm array with a 450 nm period after the PMMA removal. The inset shows a close-up of the surface, where torus-shaped features correspond to individual AgNCs. (d) An example SEM image showing a section of an AgNC array with a 450 nm period. The sizes of the DF and SEM images are 41.6 × 44.2 and 4.0 μm × 4.0 μm, respectively.

## Conclusions

We demonstrate that electrically driven actuation of metal nanoparticles tethered to metal films with DNA molecules allows the realization of spatially reconfigurable, centimeter-scale dynamic nanoparticle-on-mirror plasmonic surfaces. The fabrication process is straightforward and relies on simple methodology (thin film deposition, spin coating, and drop-casting) and readily available materials (ssDNA strands, metal nanoparticles). Fabricated eNPoMs operate with extremely low switching energy and exhibit excellent modulation of optical responses with up to 211% relative change in reflectivity at 550 nm with minimal signal degradation. When comparing to other published active NPoM systems,^6,32,33,53–56^ typically our eNPoM has comparable switching energy per cube (0.6 fJ range) and contrast of modulation (20%) compared to literature (∼fJ range, 20–50%) but the switching speed is slower than systems with optical stimuli (∼THz), comparable to electric field induced motion (∼1–10 kHz) and faster than systems based on change of a molecular state (0.1–1 Hz). Furthermore, our approach to eNPoM fabrication is compatible with various surface patterning techniques, enabling the generation of multiplexed active plasmonic surfaces and arrays of actuating nanoparticles. While we utilize photolithography and EBL in this study, other methods, e.g., nanoimprint lithography^57^ could be used to achieve similar patterning results.

The eNPoMs described here constitute fast, stable, and tunable broadband absorbers fabricated via a scalable assembly process. This fabrication process may be beneficial for applications requiring curved surfaces^58^ difficult to process with other methods, in systems utilizing interactions between DNA and biomolecules, e.g., biosensing^59^ or as tunable hot spots for dynamic fluorescence or Raman enhancements. Broader utility might require tuning to different wavelengths, achievable with DNA-coated silica particles,^60^ silica-coated DNA origami structures,^61^ or core–shell nanoparticles.^62^ The reflectance modulation demonstrated here is centered at ∼550 nm and is predominantly an intensity modulation rather than a spectral shift, because the flexible DNA tethers produce a broad distribution of gap distances that smears the ensemble resonance. Achieving resolvable spectral shifts that would enable dynamic color switching requires narrowing this distribution, for example, through rigid DNA origami spacers that constrain particle orientation and gap distance. Full visible-gamut coverage from gap distance modulation alone is further limited by the spectral range accessible for a given particle size and material. Independent RGB modulation would additionally require multiple particle populations or spatially addressable electrode geometries.

Polarization-dependent tunable filters or sensors may be realized using eNPoMs from oriented anisotropic nanoparticles. We anticipate that the approach can also be extended to more organized surface assemblies using meniscus-guided self-assembly,^33^ template confinement,^50^ chemical liftoff lithography,^63^ or DNA origami lattices.^64,65^ Further combination of the eNPoM with other device architectures or chemistries may offer higher and faster signal modulation, narrower spectral response, and more sensitive detection mechanisms for real-world applications. In this respect, one promising candidate is the DNA origami technique.^66–68^ Future efforts involve DNA origami and periodically patterned substrates^64,69^ toward tunable nanoelectromechanical metasurfaces and dark field and Raman spectroscopy sensing applications with single-molecule sensitivity.

## Methods

### Silver Nanocubes (AgNCs) Synthesis and Characterization

AgNCs were prepared using previously established methodology,^70,71^ and a detailed synthesis protocol is presented in the Supporting Information. Briefly, 20 mL of ethylene glycol (EG) in a flask was heated to 150 °C in an oil bath while stirred at 300 rpm. After 40 min, 240 μL of sodium hydrosulfide hydrate (NaSH·xH_2_O, 3 mM in EG, 56.06 g mol^-1^ , Sigma-Aldrich) was added to the flask. One minute later, in consecutive order, 2.1 mL of hydrochloric acid (HCl, 3 mM in EG) and 5 mL of poly­(vinylpyrrolidone) (PVP, 20 mg mL^–1^ in EG, 55,000 g mol^–1^) were added. After 2 min, 1.6 mL of silver trifluoroacetate (CF_3_COOAg, 282 mM in EG) was added. After 2 h, the reaction was completed, and the flask was cooled in an ice bath. Synthesized silver nanocubes were washed by centrifuging and stored in Type 1 water at 4 °C.

UV–vis absorption spectra of synthesized AgNCs were measured in a 10 mm cuvette using a spectrometer (BioSpectrometer, Eppendorf). The size and the shape of AgNCs were characterized using a transmission electron microscope (TEM FEI Tecnai). Drop-cast samples on a carbon-coated copper grid were imaged at 120 kV, and ImageJ Fiji was used to determine the average edge length of the AgNCs (Figure S5).

### Functionalizing Gold Surfaces with DNA Oligos and Mercapto-Hexanol

Nonpatterned gold surfaces were fabricated by depositing 5 nm of titanium and 50 nm of gold on silicon wafers using a physical vapor deposition system (Angstrom Engineering Inc.). We employed standard UV lithography processes to pattern the “Aalto logo” on silicon wafers (see more details in the method section “Patterning eNPoM substrate” and Supporting Information) and evaporated the same 5 nm Ti and 50 nm Au layers. In both cases, the wafers were diced into 30 mm × 10 mm chips. Before use, both nonpatterned and patterned chips were rinsed with acetone and isopropanol, dried under N_2_, and plasma cleaned for 2 min. A DNA and 6-mercapto-1-hexanol (MCH) solution was prepared by first mixing 15 μL of Type-1 H_2_O, 15 μL of 100 μM substrate strand, and 15 μL of Tris­(2-carboxyethyl)­phosphine (TCEP) in a 5 mL plastic tube, then incubating the solution for 1 h at RT. Then, 2660 μL of Type-1 water and 296 μL of 10 mM MCH were added to the solution, and Au chips were placed inside the tube and incubated for at least 24 h.

### DNA Coating of Silver Nanocubes

Our modified DNA coating protocol is based on previously reported methodology.^72,73^ Before use, the AgNCs were sonicated for 1 min 8874 μL of Type-1 water, 1050 μL of 0.2% sodium dodecyl sulfate (SDS), 127.5 μL of AgNCs stock solution (100 nM), and 400 μL of ssDNA solution were added to a 15 mL plastic tube and mixed thoroughly. The ssDNA solution contained 360 μL of Type-1 water, 36 μL of passivating ssDNA (1 mM), and 4 μL of anchor strand (100 μM). The mixture was pipetted into ten 1.5 mL tubes and frozen overnight. After thawing, the AgNCs were purified by centrifugation at 16,000 rcf for 11 min, then the supernatant was removed, and the AgNCs were resuspended in 0.02% SDS. This process was repeated four times, and after the fourth spin, the solutions were concentrated and collected into a single tube. After purification, the UV–vis absorption spectra were measured, and the concentrations were calculated (Figure S25, see Supporting Information). The concentrated DNA-coated AgNCs were stored at 4 °C.

### Attachment of AgNC on the DNA-MCH-Au Surface

After 24 h of incubation, the DNA-MCH-Au surface was rinsed with 3 × 100 μL of Type-1 water. Excess liquid was blotted away from the surface and the backside of the chip using a disposable lab wipe. Either a 100 μL solution of AgNCs or AuNCs in 0.5 × TE and 100 mM NaCl was added to the surface and incubated for 1.5 h. The concentrations of AgNCs and AuNCs during deposition were 6.8 nM and 0.4 nM, respectively. After deposition, the gold film was rinsed with 3 × 100 μL of 0.5 × TE and 100 mM NaCl buffer. The samples were stored in the same buffer in a fridge at 4 °C. After optical characterization, the surfaces of eNPoMs were characterized using scanning electron microscopy, and fill fractions and average cube-to-cube distance were calculated (Figures S26 and S27).

### DC Voltage-Biased Reflectance Measurements

Samples were loaded into a liquid cell consisting of a plexiglass cover and base with a polydimethylsiloxane (PDMS) spacer (see Figure S28). The cell was filled with buffer and placed in a spectrophotometer (V-770, Jasco) equipped with an absolute reflectance unit (ARSN-917, Jasco). The gold surface and indium tin oxide (ITO) window were connected to the function generator. During reflectance measurements, we cycled between negative and positive voltages to confirm that samples were active and reversible. After a few steps of cycling were recorded, the buffer was exchanged. The order of measured buffers was from 100 mM to 0 mM NaCl.

### High-Speed Optical Measurements

Encapsulated samples were fabricated for switching speed measurements by forming a liquid cell between an Au-coated silicon chip (15 mm × 15 mm) and an ITO-coated glass slide using a 3 mm diameter 120 μm thick imaging spacer (Grace BioLabs SecureSeal). We illuminated the sample using a xenon lamp with a 532 nm bandpass filter and epi-illumination through a ×10 brightfield objective in an upright microscope. We captured the reflected light by focusing it on an amplified silicon photodetector (Thorlabs PDA-100A2). One million data points were measured at each driving frequency while cycling the sample through a range of frequencies from 10 Hz to 50 kHz. For each frequency, the data from each cycle was then normalized and averaged to obtain an average cycle. The switching amplitude was then extracted from this average cycle. Electrochemical impedance of the encapsulated cell was measured using a potentiostat (Interface 1010T, Gamry Instruments) at the open circuit potential of the cell using a 10 mV amplitude.

### Finite Element Method Simulation of Nanoparticle-On-Mirror System

Ansys HFSS simulation software was used to simulate the reflectance of silver nanocubes on top of 50 nm gold and 5 nm titanium films within a periodic unit cell. The surrounding medium was water (refractive index = 1.33). The DNA layer between the nanocube and the gold surface is assumed to be sparse and effectively estimated to have the same refractive index as water. The gap distance between AgNC and gold film varied between 5 and 25 nm, and the particle size between 50.4 and 61.4 nm. We calculated a normalized reflectance curve for each gap size by accounting for the particle size distribution and averaging reflectance curves across different particle sizes with weight factors (see Supporting Information). The simulated reflectance spectra were multiplied twice by the transmission spectrum of the ITO window to match experimental data better, since the detected signal passes through ITO upon entry to the liquid cell and after reflection from the gold film.

### Patterning eNPoM Substrates

The “Aalto logo” was patterned on silicon wafers using standard UV lithography methods. AZ 5214E (Microchemicals GmbH) photoresist was spin-coated to a thickness of 1.5 μm on the silicon wafer and baked at 90 °C for 1 min, and the Aalto logo pattern was exposed using UV maskless lithography (Heidelberg Instruments MLA150, 405 nm). The exposed pattern was developed using AZ 351B developer for 90 s, followed by a 90 s wash in deionized water. The wafer was dried, and 5 nm of titanium and 50 nm of gold layers were evaporated on top. Liftoff was performed by immersing the metal-coated wafer in acetone for 2 h at room temperature. The wafer was rinsed with acetone and IPA to remove any residual metal from the surface.

The eNPoM arrays were fabricated by spin coating a PMMA 950 A2 layer (thickness approximately 100 nm) on top of the gold film and baking the samples for 90 s at 180 °C. Hole array masks were patterned using electron beam lithography. After patterning, samples were developed in 3:1 = isopropanol (IPA): methyl isobutyl ketone (MIBK) for 60 s, rinsed using IPA, and N_2_ dried. After development, the samples were plasma cleaned using Tergeo plasma cleaner (Air (10 sccm), water (10 sccm), 10 s, 75 W). The surface functionalization with DNA and MCH was done the same way as before (i.e., subsection Functionalizing gold surfaces with DNA oligos and mercapto-hexanol). Afterward, samples were dipped twice in Type-1 water, and AgNCs were drop-cast for 1 h and 20 min. The AgNCs concentration was 60 nM during drop-casting, and the buffer contained 0.1 M NaCl, 0.1% SDS, and 2 mM MgCl_2_. After drop-casting, the surface was rinsed with Type-1 water, dipped in an 80% IPA and 20% acetone solution, and then placed in a 60% IPA and 40% acetone solution for PMMA removal (30 min, 40 °C). Then, the samples were dipped once in a 0.5 × TE and 0.1 M NaCl buffer and then stored in the same buffer. For optical microscopy characterization of eNPoM arrays, we utilized a self-made liquid cell and an upright microscope (Olympus BX53). We applied an AC square voltage with 100–200 mV peak amplitude between the ITO and the gold film and recorded optical images. Afterward, samples are rinsed once with 300 μL Type-1 water and N_2_-dried for SEM characterization.

## Supplementary Material















## Notes

Reflectance spectra of different eNPoM PSs under DC voltage and AC square wave excitation, TEM images and UV–vis spectra of different AgNCs batches and the corresponding edge length distributions, numerical simulations of individual AgNC on a gold film including, voltage versus time curves for a blank sample containing thin gold and titanium films showing the charging time, electrochemical impedance measurement of eNPoM PSs, intensity versus time curves of different eNPoM PSs with the data acquired either using a color camera or a high-speed camera, color images of the “Aalto Logo” eNPoM and derived Fourier transform maps from the same images, Fourier phase maps of the “Aalto Logo” eNPoM and associated phase change curves along designated lines, SEM images of AgNCs drop-casted on PMMA hole arrays and the related fill fractions plots, dark field and SEM images of AgNC arrays and associated fill fraction plots and areal switching uniformity analysis, SEM images of different eNPoM PSs, a schematic view of the liquid cell (PDF)
Video S1 showing actuation of eNPoM surface using an alternating square wave excitation with 300 mV peak amplitude and 1 Hz frequency (AVI)
Video S2 showing actuation of eNPoM surface using an alternating square wave excitation with 300 mV peak amplitude and 10 Hz frequency (AVI)
Video S3 showing actuation of eNPoM surface using an alternating square wave excitation with 300 mV peak amplitude and 100 Hz frequency (AVI)
Video S4 showing actuation of eNPoM surface using an alternating square wave excitation with 300 mV peak amplitude and 1000 Hz frequency (AVI)
Video S5 demonstrating multi-input actuation of Aalto logo pattern (AVI)
Video S6 showing actuation of 40 μm × 40 μm arrays of AgNCs with different periods (AVI)
