# Supplementary material for: Active Plasmonic Surfaces via Electrically Driven Actuation of DNA-Tethered Nanoparticles
Source: ACS Nano. 2026 Jun 9;20(24):17663–73. doi: 10.1021/acsnano.6c04919 (PMC13296604; doi:10.1021/acsnano.6c04919)
Supplement: Supplementary file 1 [file nn6c04919_si_001.pdf]

Supplementary information for “Active plasmonic surfaces *via* electrically driven actuation of DNA-tethered nanoparticles”

Mohammed M. A. Al Hussain[1*], Abraham Kipnis[1*], Anna Lumppio[1], Narat Witwiyaruj[1], Sesha Manuguri[1], Xuan-Hung Pham[1], Pierre Bléteau[1], Maxime Fauconnier[1], Mohammadmahdi Asgari [2], Viktar Asadchy[2], Anton Kuzyk[1], Kosti Tapio[1]

[1] Department of Neuroscience and Biomedical Engineering, Aalto University, Espoo, 02150, Finland.

[2] Department of Electronics and Nanoengineering, Aalto University, Espoo, 02150, Finland.

*Equal contribution

1. DC voltage-biased reflectance data

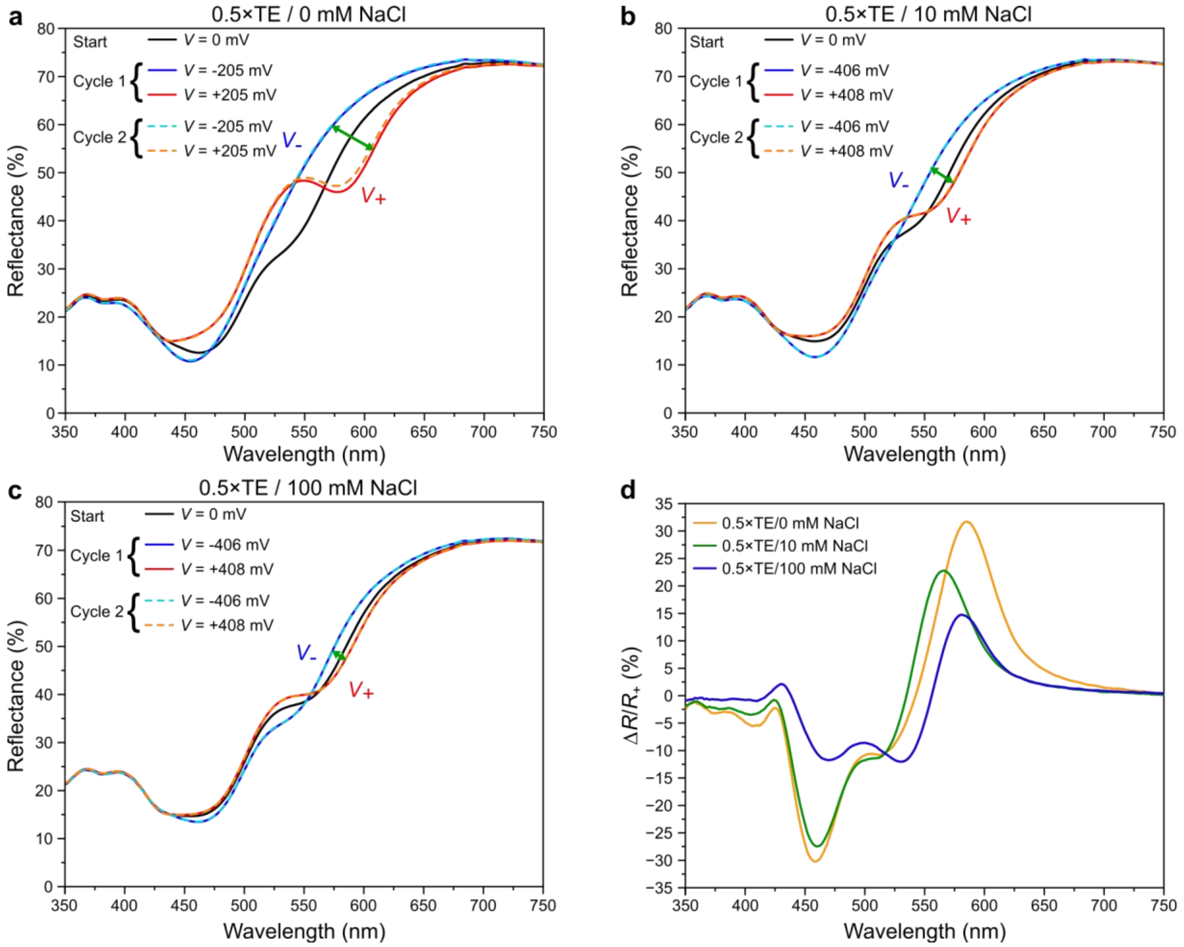

Figure S1. Reflectance measurements of AgNC eNPoM, where the “anchor” strand length is 12 nt. (a)-(c) The reflectance curves of the eNPoM surface in different buffer conditions when voltages are cycled twice between positive and negative values. (d) The relative change in reflectance $\Delta R/R_{+}$ in different buffer conditions calculated from the curves in a-c.

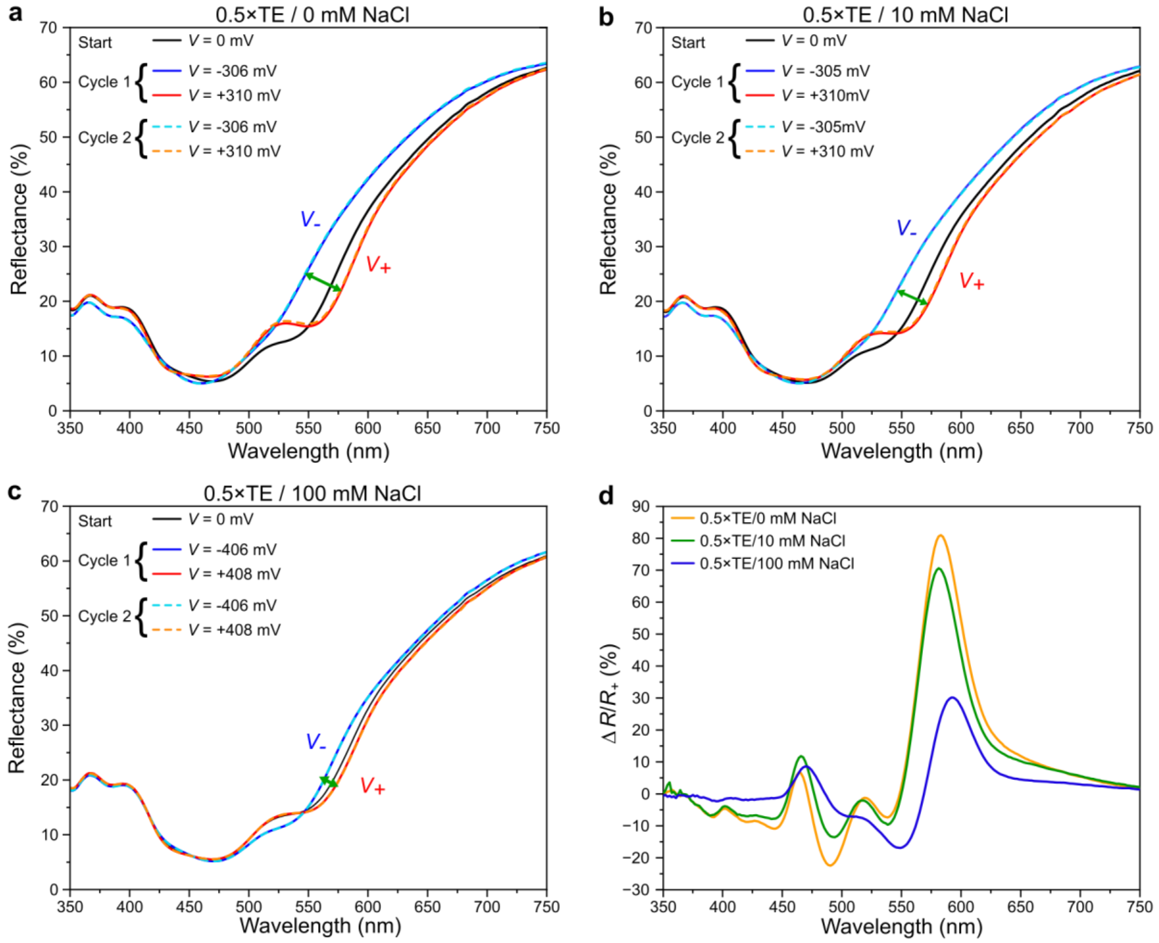

Figure S2. Reflectance measurements of AgNC eNPoM, where the "anchor" strand length is 20 nt. (a)-(c) The reflectance curves of the eNPoM surface in different buffer conditions when voltages are cycled twice between positive and negative values. (d) The relative change in reflectance $\Delta R/R_{+}$ in different buffer conditions calculated from the curves in a-c.

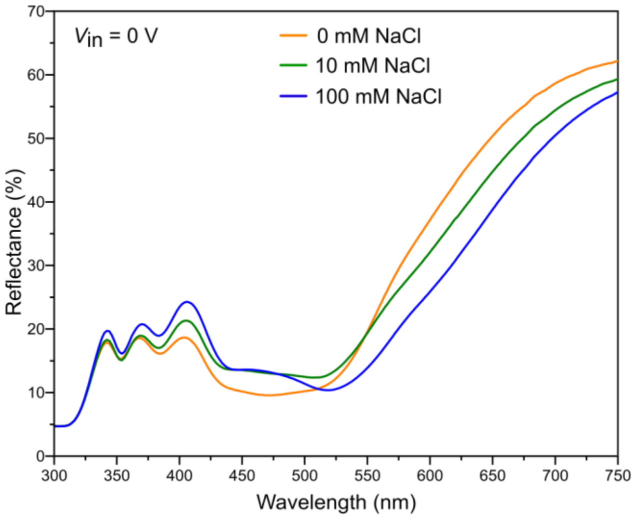

Figure S3. Reflectance baseline shift of AgNC eNPoM in different buffer conditions, where the "anchor" strand length is 26 nt. The curves are the 0 V bias curves from Figure 2.

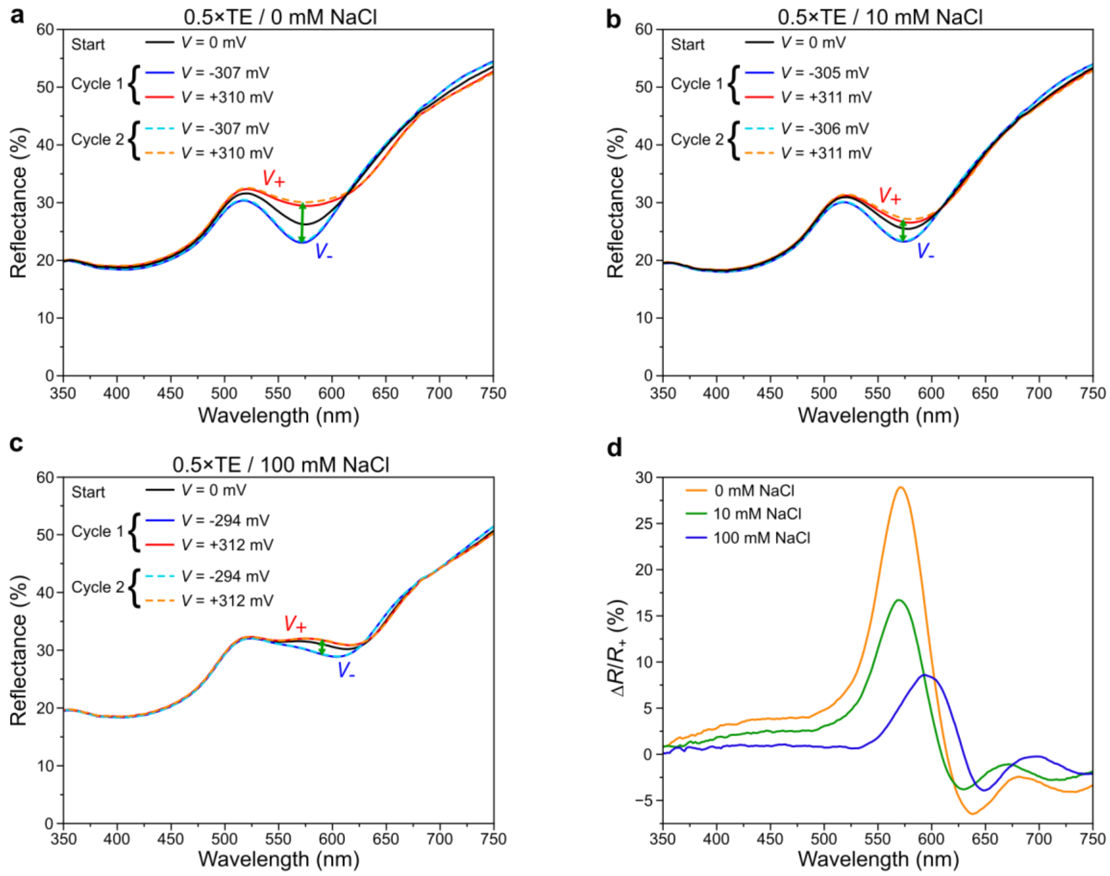

Figure S4. Reflectance of AuNCs eNPoM, where the "anchor" strand length is 26 nt. (a)-(c) The reflectance curves of the eNPoM surface in different buffer conditions when voltages are cycled twice between positive and negative values. (d) The relative change in reflectance $\Delta R/R_{+}$ in different buffer conditions calculated from the curves in a-c.

2. Characterization of AgNC

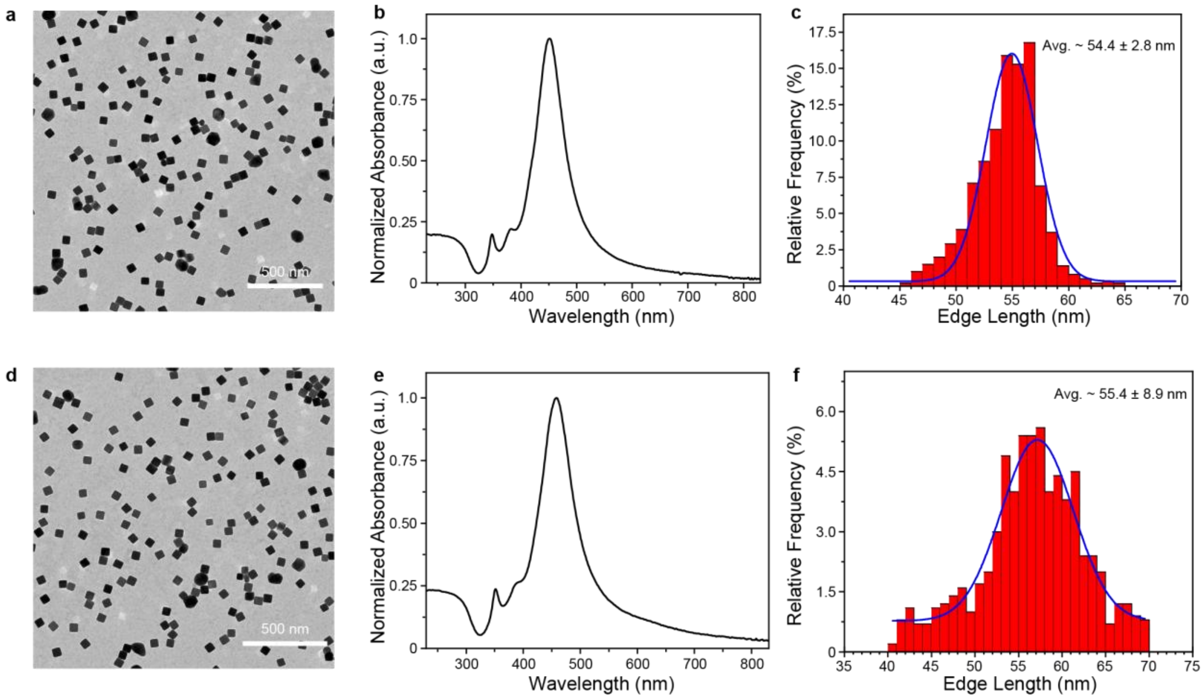

Figure S5. Characterization of AgNCs. (a) A representative TEM image of AgNCs with an absorbance peak at 452 nm. (b) Normalized absorbance of the AgNCs in a measured using a UV-Vis spectrophotometer. (c) Estimated edge length relative frequency of AgNCs from a. The standard deviation and the average size of 1000 measured cubes are shown (inset). (d) A representative TEM image of AgNCs with 460 nm absorbance. (e) Normalized absorbance of the 460 nm AgNCs measured using a UV-Vis spectrophotometer. (f) Estimated edge length relative frequency of AgNCs from d. The standard deviation and the average size of 780 measured cubes are shown (inset). The blue curves in c and f represent the Gaussian distribution.

3. Finite element method simulations of AgNC on the Au surface

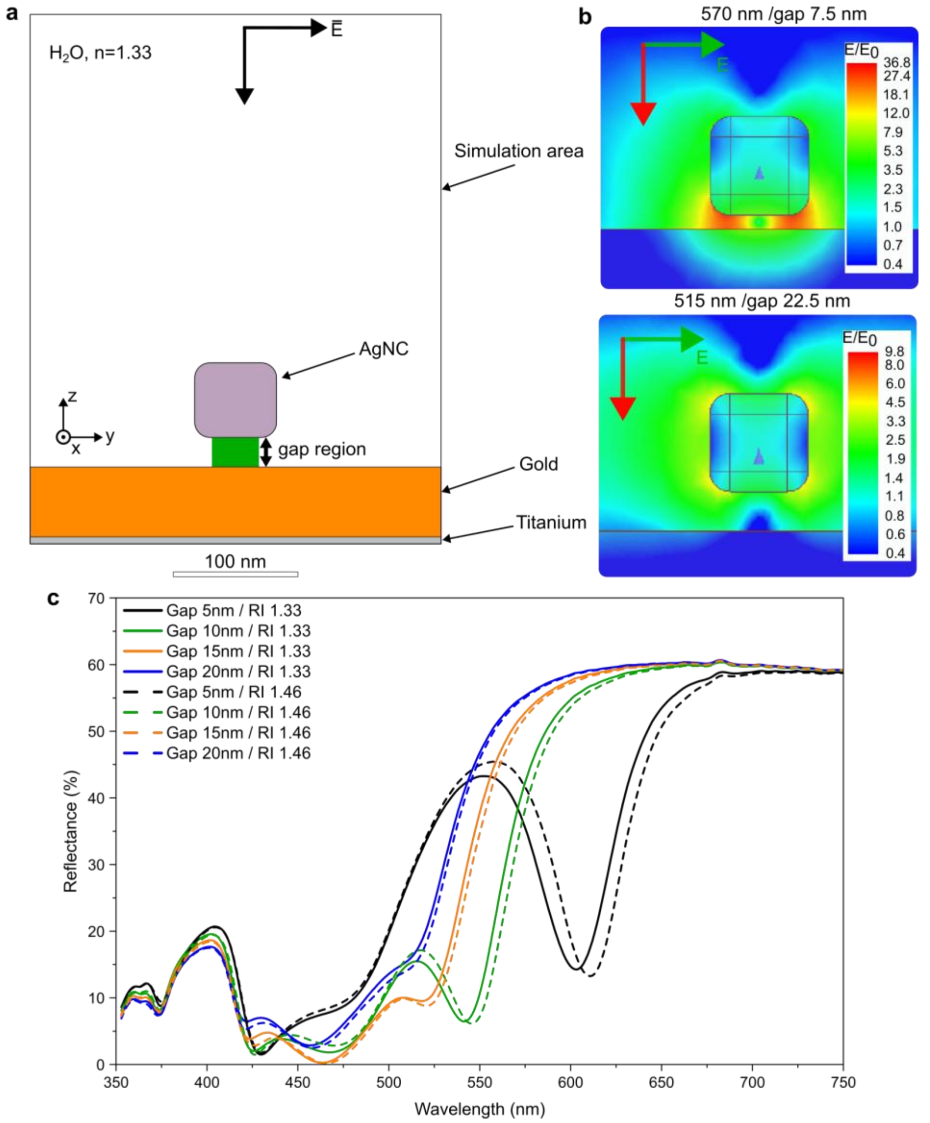

Figure S6. The schematic view of the used simulation model and the electric field distribution of AgNC on gold-titanium film. (a) The used simulation model consisting of a single AgNC surrounded by periodic boundary conditions on top of 50 nm gold and 5 nm titanium films. The gap distance and AgNC size are varied during the simulations. (b) Total electric field distributions of 55.4 nm AgNC on top of the gold film, when the gap and the excitation wavelength are 7.5 nm and 570 nm (top image) or 22.5 nm and 515 nm (bottom image), respectively. The image size is 150 nm × 150 nm. (c) The simulated reflectance for 5, 10, 15 and 20 nm gap sizes, when the refractive index in the gap region is increased from 1.33 to 1.46.

The Ansys High-Frequency Simulation Software (HFSS), part of Ansys Electronic Desktop, was used to simulate the reflectance of a periodic array of AgNCs placed above 50 nm gold and 5 nm titanium films with a gap between the AgNC and the gold film (see Figure S6a). Periodic boundary conditions were applied to the four sides of the unit cell. The host medium was water (refractive index = 1.33), and the

refractive index of the gap region (the green box) is adjusted to either 1.33 or 1.46. The curvature of AgNC was 11.6 nm. The gap distance is varied between 5 and 25 nm, and the particle size is between 50.4 and 61.4 nm. The unit cell dimensions were 264 nm × 264 nm × 500 nm.

The light excitation propagates along the z-axis from a Floquet port at the top of the simulation domain. The HFSS uses adaptive meshing, with the maximum mesh size around the cube being between 0.88 – 1.1 nm, 1.35 – 1.5 nm in water, and 8.0 – 11.9 nm elsewhere. The reflectances and electric field distributions were plotted in Figures 3, S6b, and S7. Two distinct near-field electric field distributions are evident in the simulations[1]: the gap mode and the inter-particle mode. The gap mode is the dip around 550 – 600 nm corresponding to the plasmonic resonance between the particle and the Au film. The inter-particle mode is observable at large gap distances (above 15 nm) around 500 – 525 nm. An example of the gap mode electric field distribution is shown for a 7.5 nm gap at λ = 570 nm and with the gap region refractive index of 1.33 in Figure S6. When the gap increases to 17.5 nm, the coupling between two adjacent particles (the inter-particle mode due to periodic boundary conditions) starts to dominate. This can be seen in Figure S6 for a 22.5 nm gap and 515 nm illumination. Simulated spectra were multiplied by the measured transmission spectrum of the ITO window (Figure S7c) twice, since the measured signal passed through ITO twice (entry to the liquid cell and exit after reflection).

To estimate the effect of the DNA layer in the gap region to the reflectance spectrum, the refractive index in the gap region is increased to 1.46 corresponding to RI of ssDNA from Elhadj *et al.*[2], which results in red-shifts in the reflectance spectra compared to the case of 1.33 refractive index as seen in Figure S6c. Figures 3 and S7 show fits of simulated data to the 12, 20 nt and 26 nt “anchor” strand reflectance

curves from Figures 2, S1 and S2. The fits are constructed by summing the simulated curves from Figure 3 together with the appropriate fitting factors. Here, we have used a least square fitting to solve fitting factors for each simulated spectra giving the closest fit for both the negative and positive DC bias voltage curves. The solved fitting factors are shown in Figure S7d.

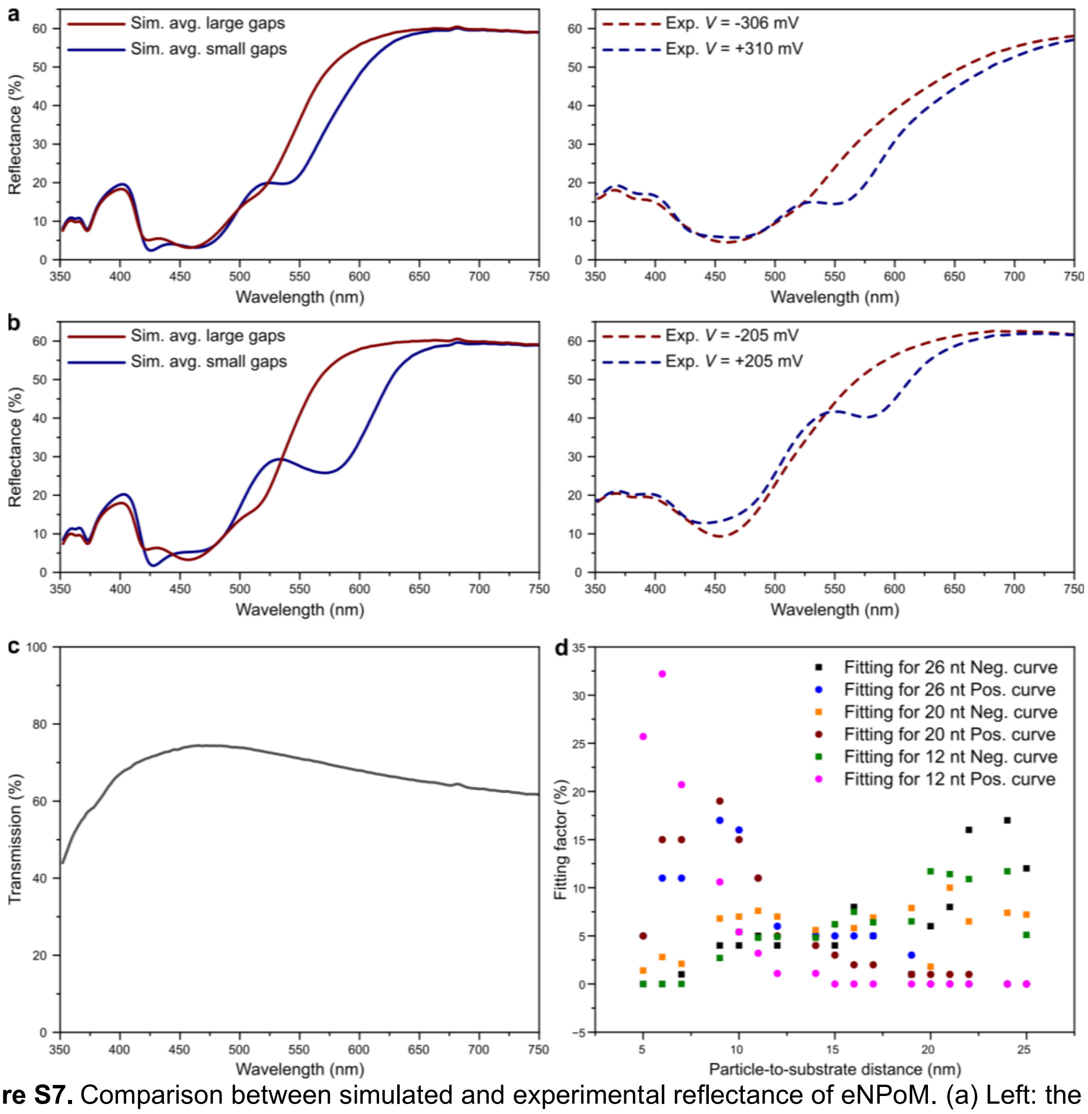

Figure S7. Comparison between simulated and experimental reflectance of eNPoM. (a) Left: the red and blue curves are constructed simulated curves for the 20 nt “anchor” strand eNPoM, where the simulated spectra from Figure 3 have been summed together with appropriate fitting factors. Right: the dashed lines are the experimental curves from Figure S2 with 0 mM NaCl. (b) Left: the red and blue curves are constructed simulated curves for 12 nt “anchor” strand eNPoM, where the simulated spectra from Figure 3 have been summed together with appropriate fitting factors. Right: The dashed lines are the experimental curves from Figure S1 with 0 mM NaCl. (c) The transmission spectrum of the ITO covered glass. (d) The fitting factors used to construct the simulated curves in Figures 3b, S7a and S7b.

4. eNPoM AC voltage actuation data

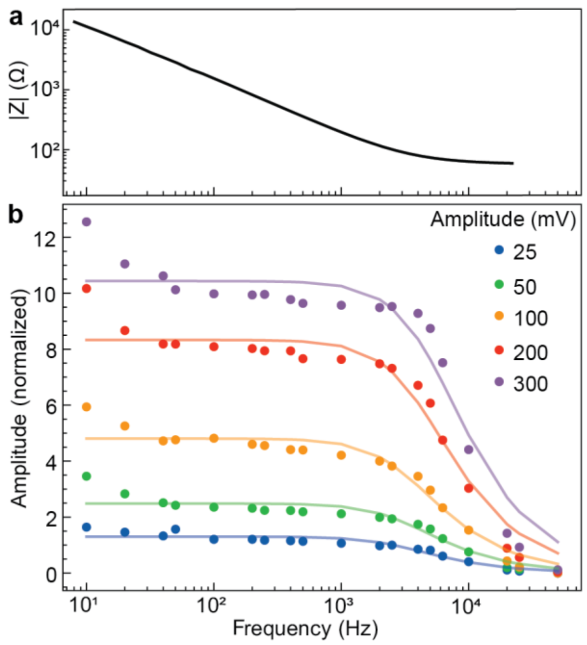

Figure S8. Ensemble frequency response of encapsulated eNPoM film in 10 mM NaCl 0.5×TE buffer. (a) Electrochemical impedance measurement of an encapsulated liquid cell. (b) Reflectance modulation amplitude response as a function of driving frequency and applied voltage. The data fit an overdamped oscillator model (solid lines).

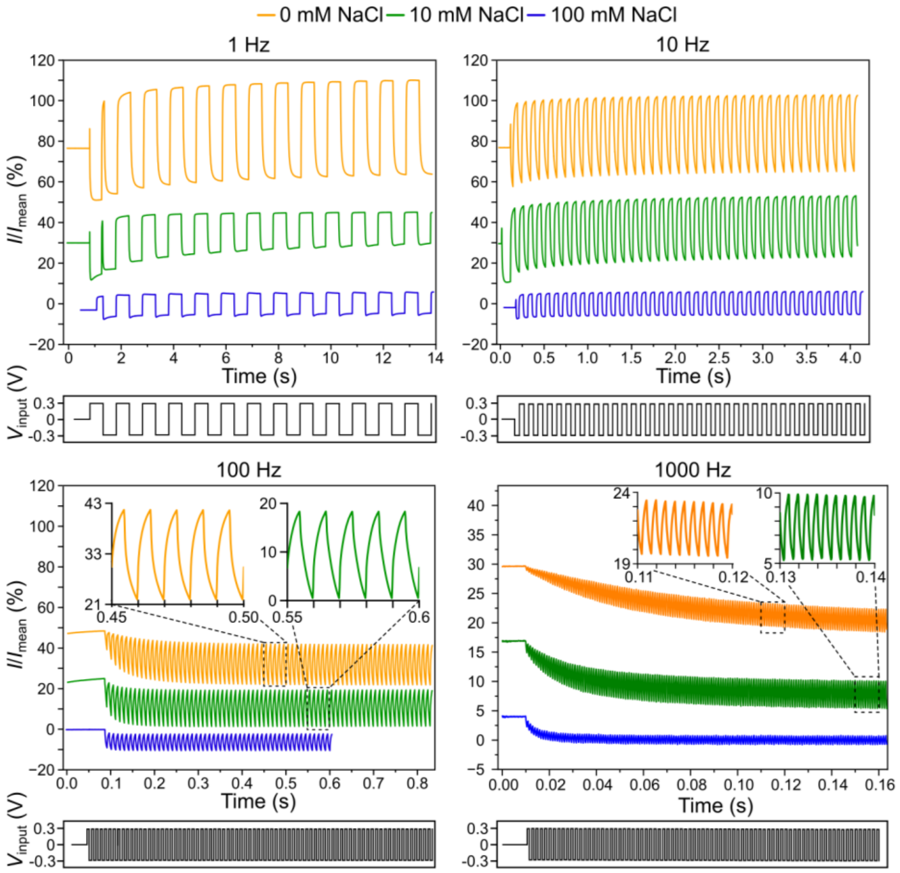

Figure S9. High-speed cycling of 26 nt "anchor" strand eNPoM switching at 1, 10, 100 and 1000 Hz. The time series data from the whole video showing multiple cycles. The 0 mM and 10 mM NaCl curves are offset for clarity. The insets in the 100 and 1000 Hz plots are snapshots from the corresponding time frame. Data was acquired from samples enclosed in the flow cell shown in Figure S28.

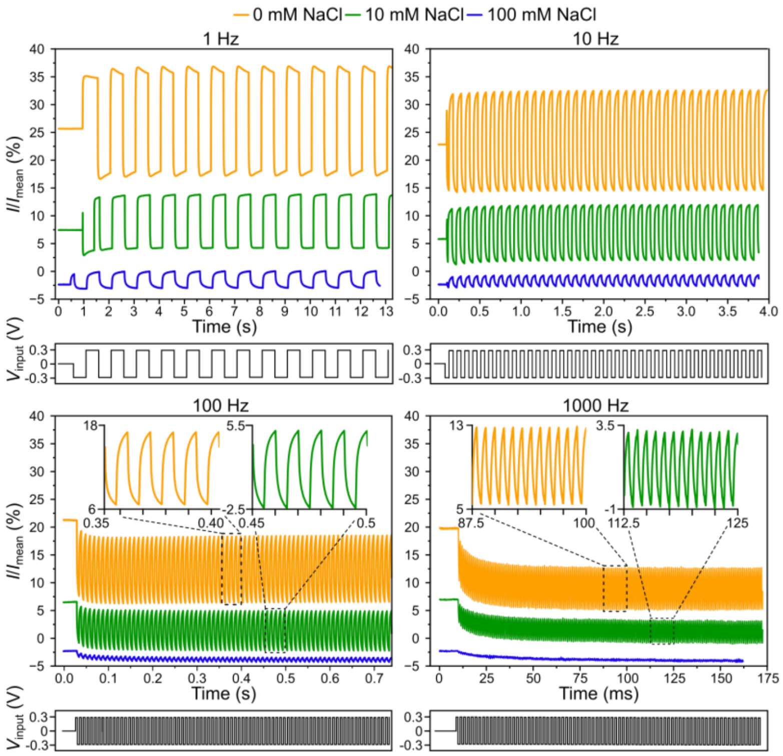

Figure S10. High-speed cycling of 12 nt “anchor” strand eNPoM. The time series data extracted from different switching videos shows multiple cycles across different buffer conditions. The 0 mM and 10 mM NaCl curves are offset for clarity. The insets in the 100 and 1000 Hz plots are snapshots from the corresponding time frame. Data was acquired from samples enclosed in the flow cell shown in Figure S28.

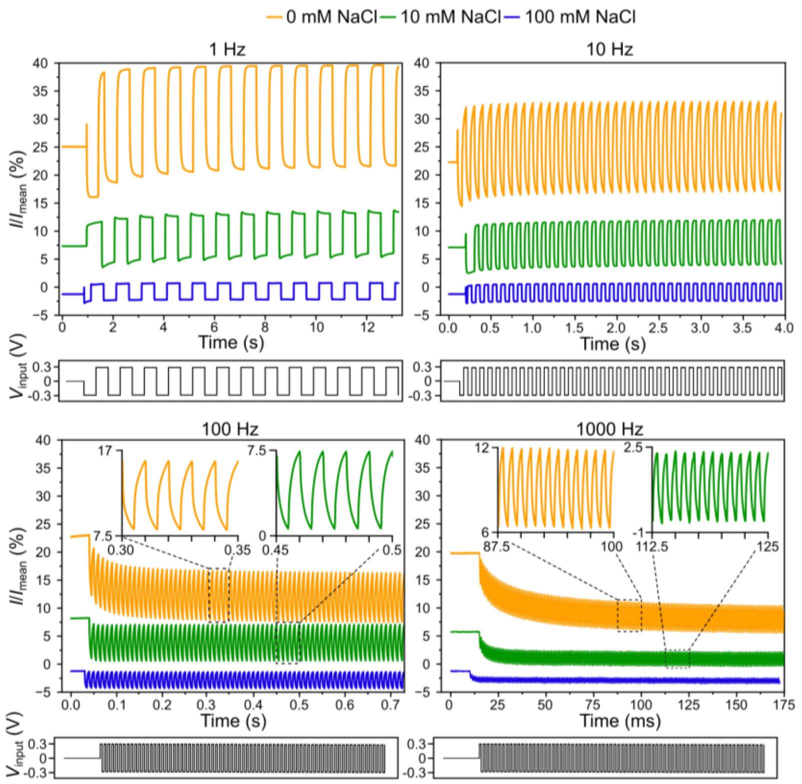

Figure S11. High-speed cycling of 20 nt "anchor" strand eNPoM. The time series data extracted from different switching videos shows multiple cycles across different buffer conditions. The 0 mM and 10 mM NaCl curves are offset for clarity. The insets in the 100 and 1000 Hz plots are snapshots from the corresponding time frame. Data was acquired from samples enclosed in the flow cell shown in Figure S28.

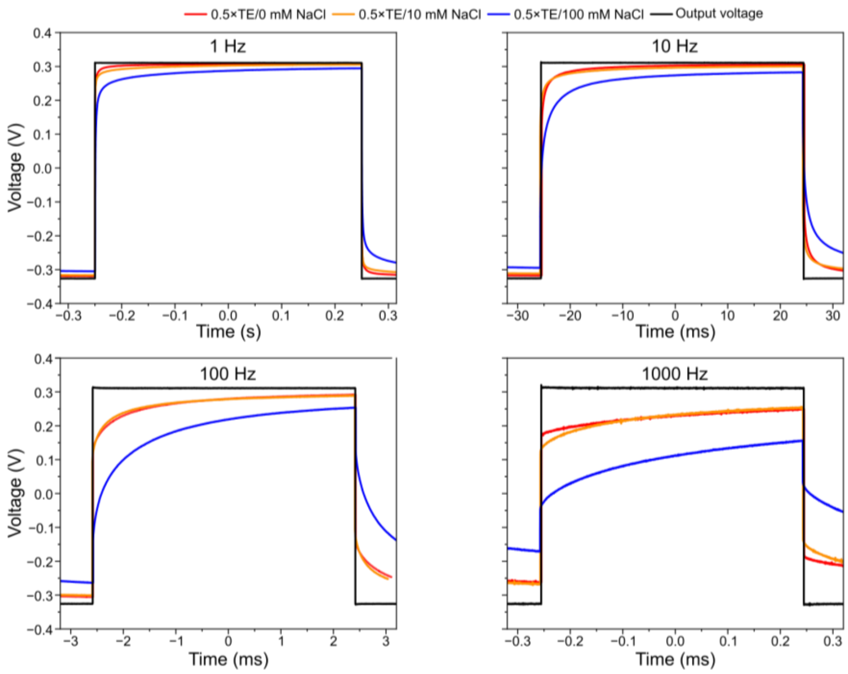

Figure S12. Frequency and buffer-dependent voltage drops over the liquid cell shown in Figure S28. The output voltage is the open-circuit voltage of the function generator.

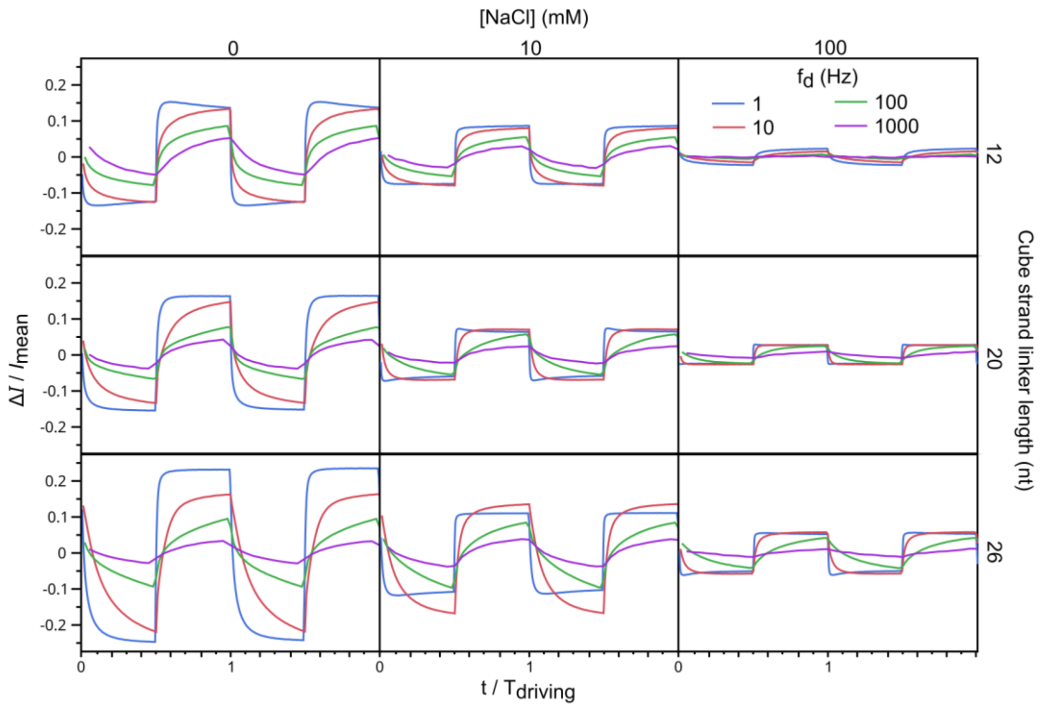

Figure S13. High-speed camera data demonstrating the sensitivity of the eNPoM to "anchor" strand length, buffer condition, and AC driving frequency f_d. Data was acquired from samples enclosed in the flow cell shown in Figure S28. The initial offset from the intensity is removed, and the resulting ΔI is normalized by the mean intensity of the data set.

5. Multioutput Aalto logo eNPoM measurements

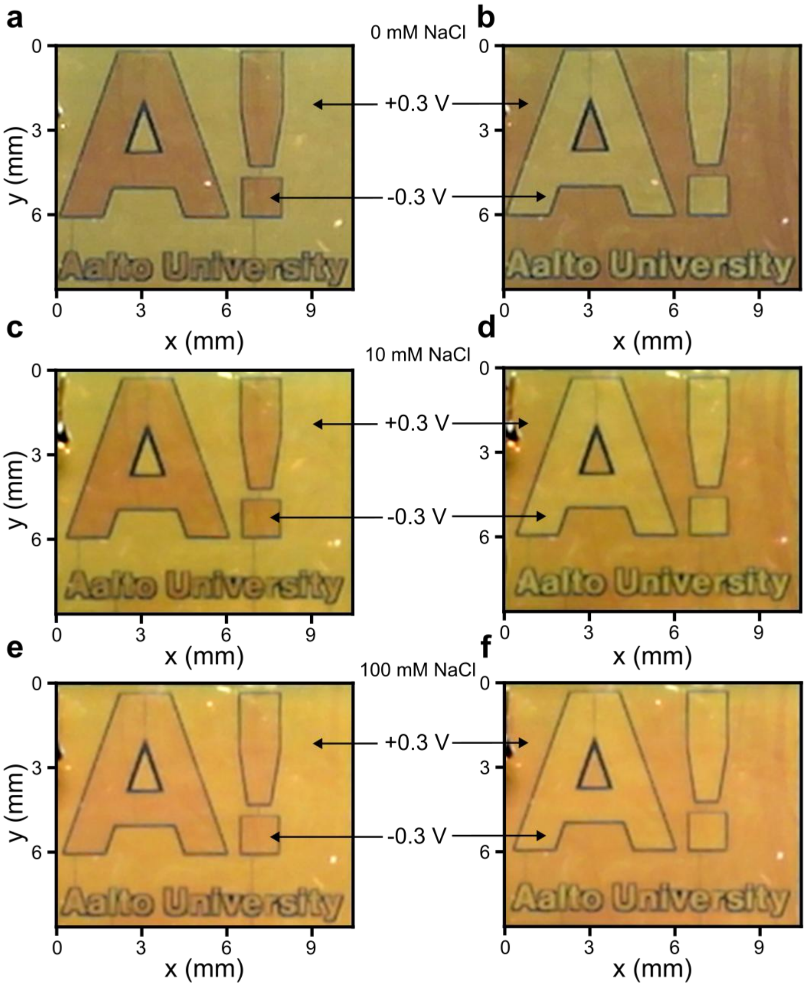

Figure S14. Cycling of multi-output "Aalto Logo" eNPoM. (a)-(f) Images of Aalto logo patterned eNPoM, where the letters and background are switching separately with a 180° phase shift. The left and right columns have a half-cycle difference between them. Each row represents different buffer conditions. Aalto logo patterned eNPoM. Credit: Aalto University. Logo used with permission.

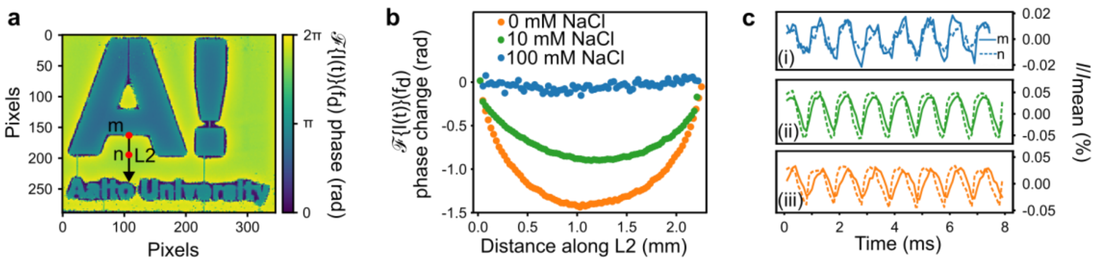

Figure S15. Demonstration of crosstalk between the letter "A" and the background in the multioutput "Aalto logo" sample at 1 kHz. (a) The intensity map shows the phase of the complex Fourier transform of the intensity for the Aalto logo sample driven at 1 kHz in 0 mM NaCl buffer conditions. (b) shows a line-cut of the data from a along the line L2 acquired with a 1 kHz AC drive frequency and in different buffer conditions. (c) The plots show the normalized intensity curves at the end (the point m, the solid line) and midpoint (the point n, the dashed line) of line L2 for (i) 100 mM NaCl, (ii) 10 mM NaCl, (iii) 0 mM NaCl, demonstrating the phase delay between regions at lower salt concentrations. Data was acquired from samples enclosed in the flow cell shown in Figure S28. Aalto logo patterned eNPoM. Credit: Aalto University. Logo used with permission.

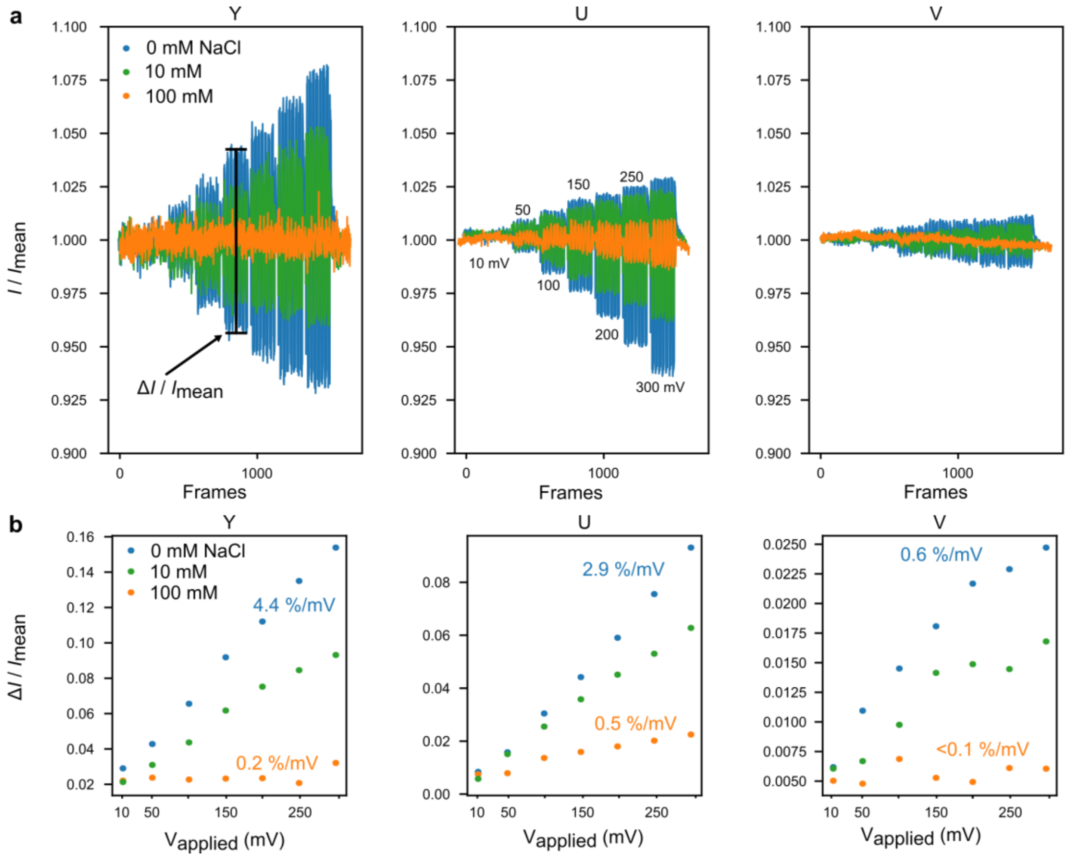

Figure S16. Characterizing the sensitivity of the eNPoM in respect to the applied electric field. The sample was illuminated, and the reflection was recorded with a color camera while the function generator was switched on and off (square wave, 1 Hz, 10 cycles) with increasing amplitude, from 10 mV to 300 mV, in steps of 50 mV. (a) The extracted Y (luma), U and V (chroma) channels from the color camera data. An example of the amplitude change $\Delta I / I_{mean}$ used in b is highlighted with the line. (b) The amplitude change of the channels $\Delta I / I_{mean}$ as a function of the applied voltage and buffer NaCl concentration. The sensitivity of the system for each channel (in units %/mV) was calculated by fitting a straight line to the data in b. Data was acquired from samples enclosed in the flow cell shown in Figure S28.

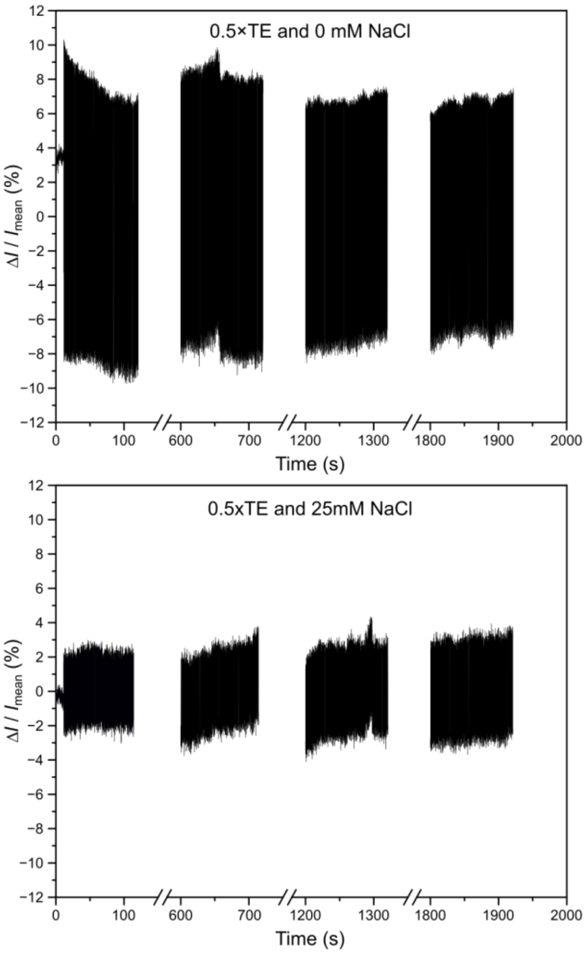

Figure S17. The reliability test of the multioutput "Aalto logo" sample. The intensity of the letter "A" is analyzed over 32 min, where the voltage is applied the whole time and the intensity is recorded at the time intervals of 0-2, 10-12, 20-22 and 30-32 min. The initial offset from the intensity is removed, and the resulting Δ*I* is normalized by the mean intensity of the analyzed data set. Data was acquired from samples enclosed in the flow cell shown in Figure S28.

6. Characterizing patterned eNPoM substrates

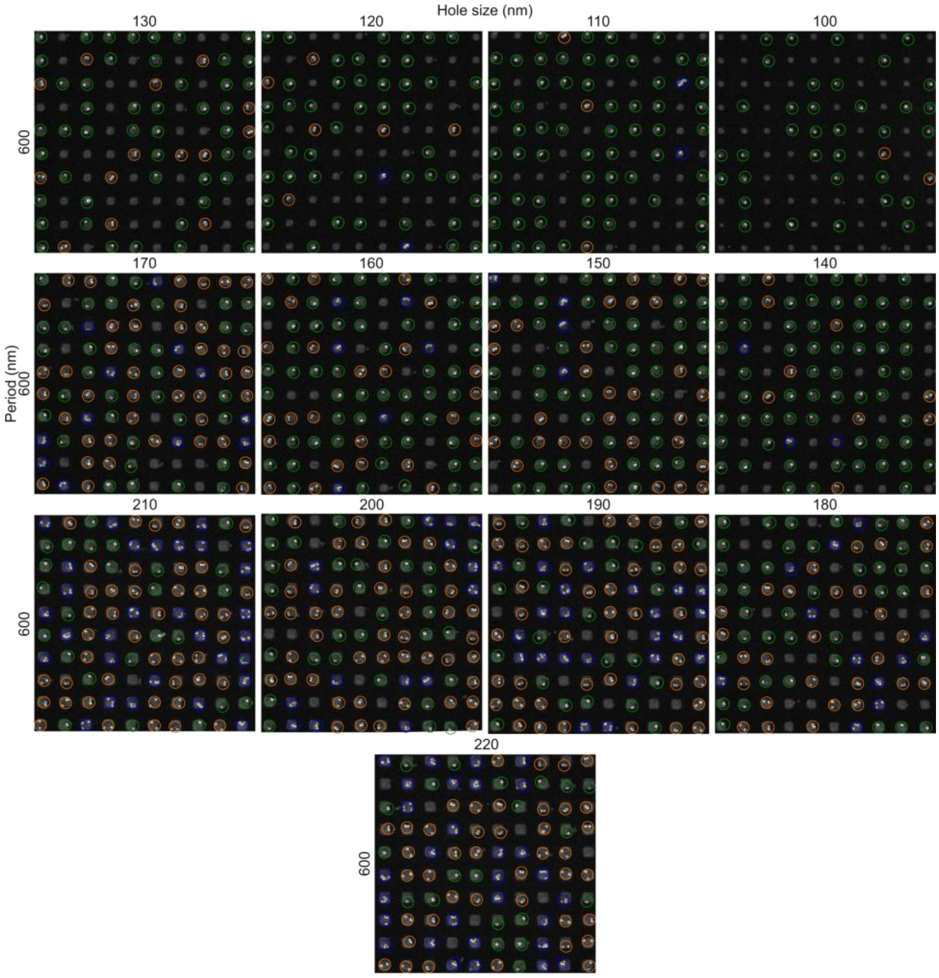

Figure S18. SEM images of 10 × 10 PMMA hole arrays with a 600 nm period and different hole sizes. The green, orange, and blue circles correspond to monomers, dimers, and aggregates, respectively.

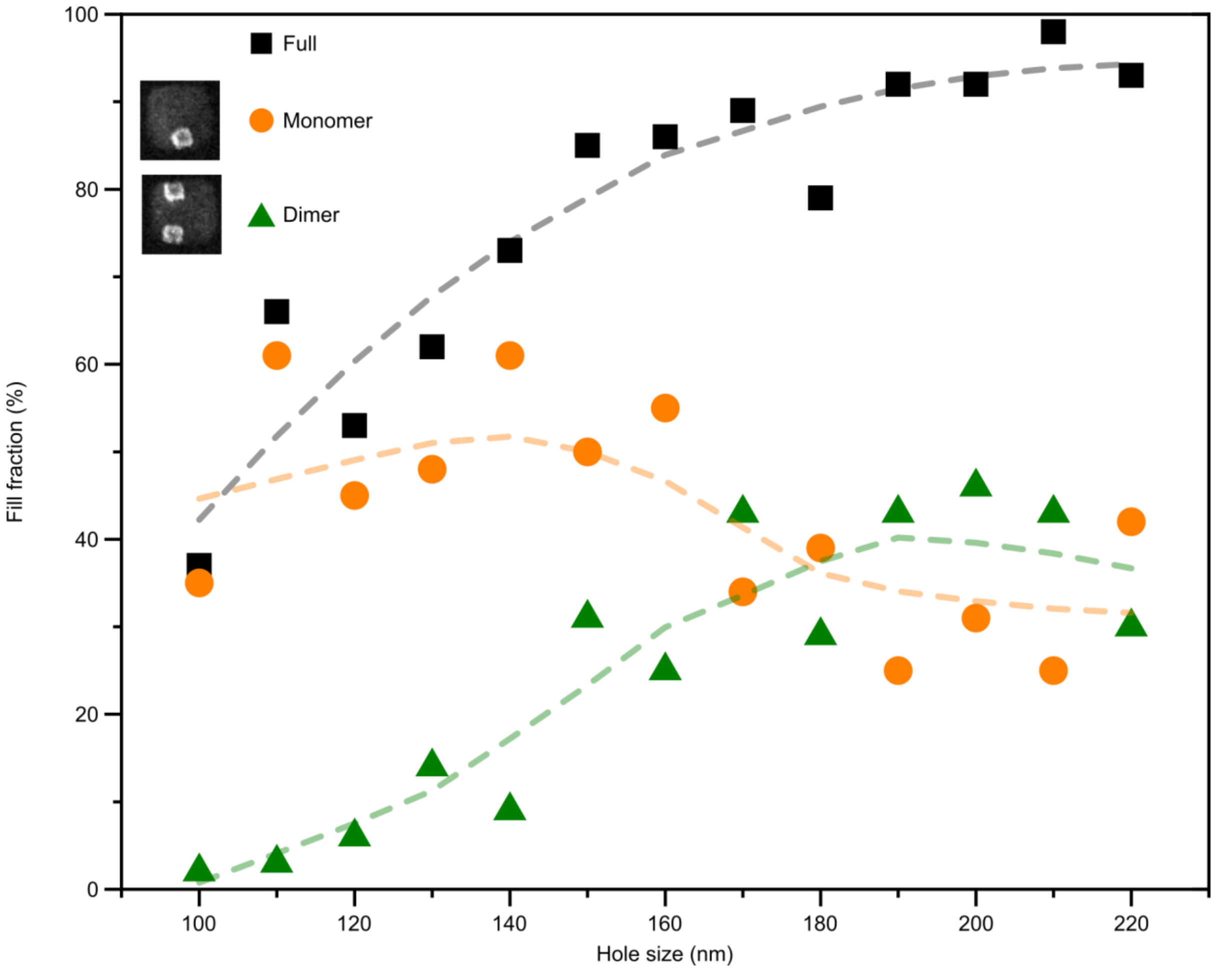

Figure S19. The hole size-dependent fill fractions for monomers, dimers and non-empty holes calculated from Figure S18. The dashed lines are plotted for visualization purposes.

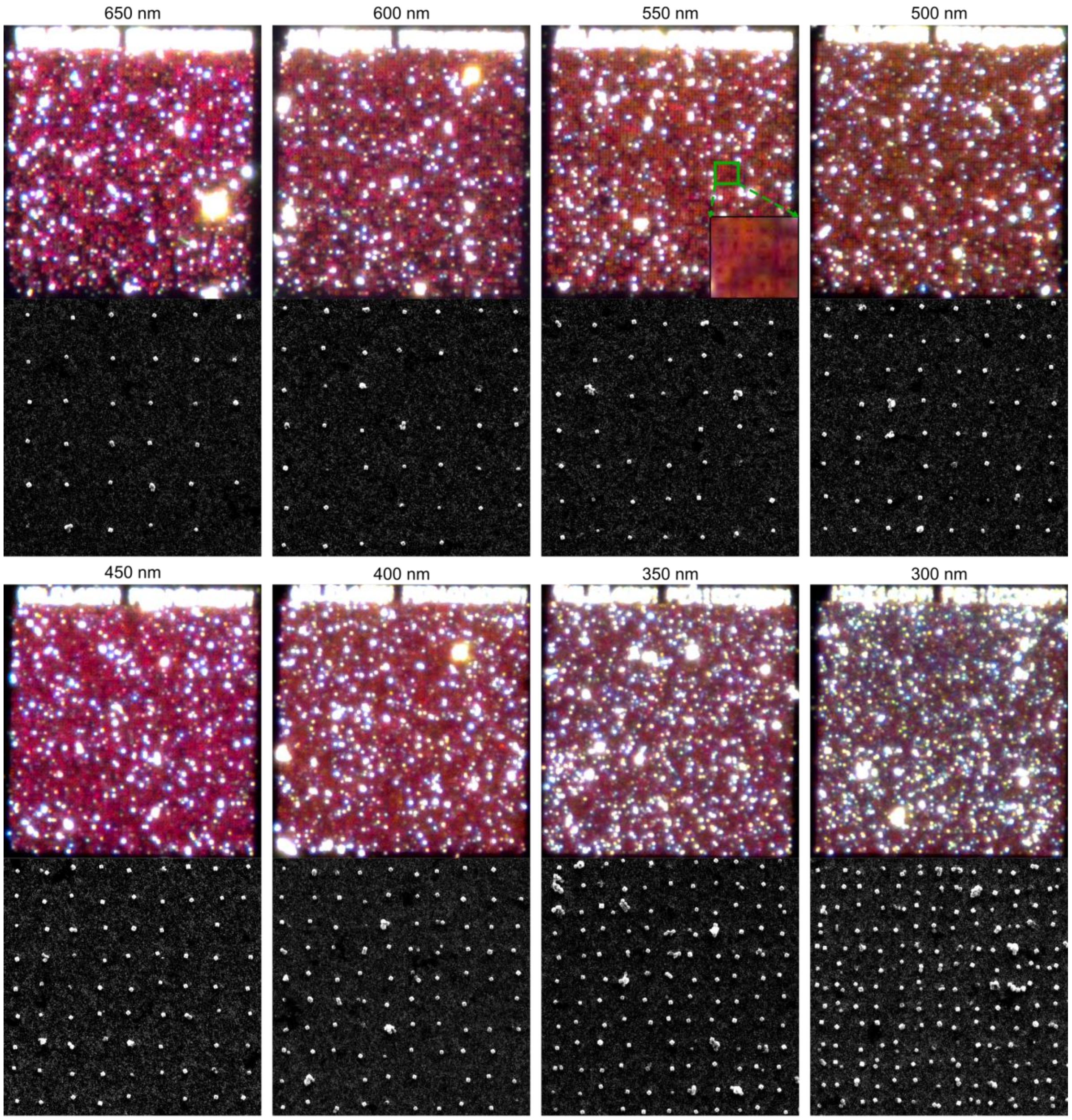

Figure S20. An example of patterned AgNC arrays with different periods. The array period is listed at the top of the dark field image. The arrays in DF images are 41.6 μm x 44.2 μm. The sizes of the SEM images are 4 μm × 4 μm. The inset in the 550 nm array shows torus-shaped objects corresponding to single AgNCs.

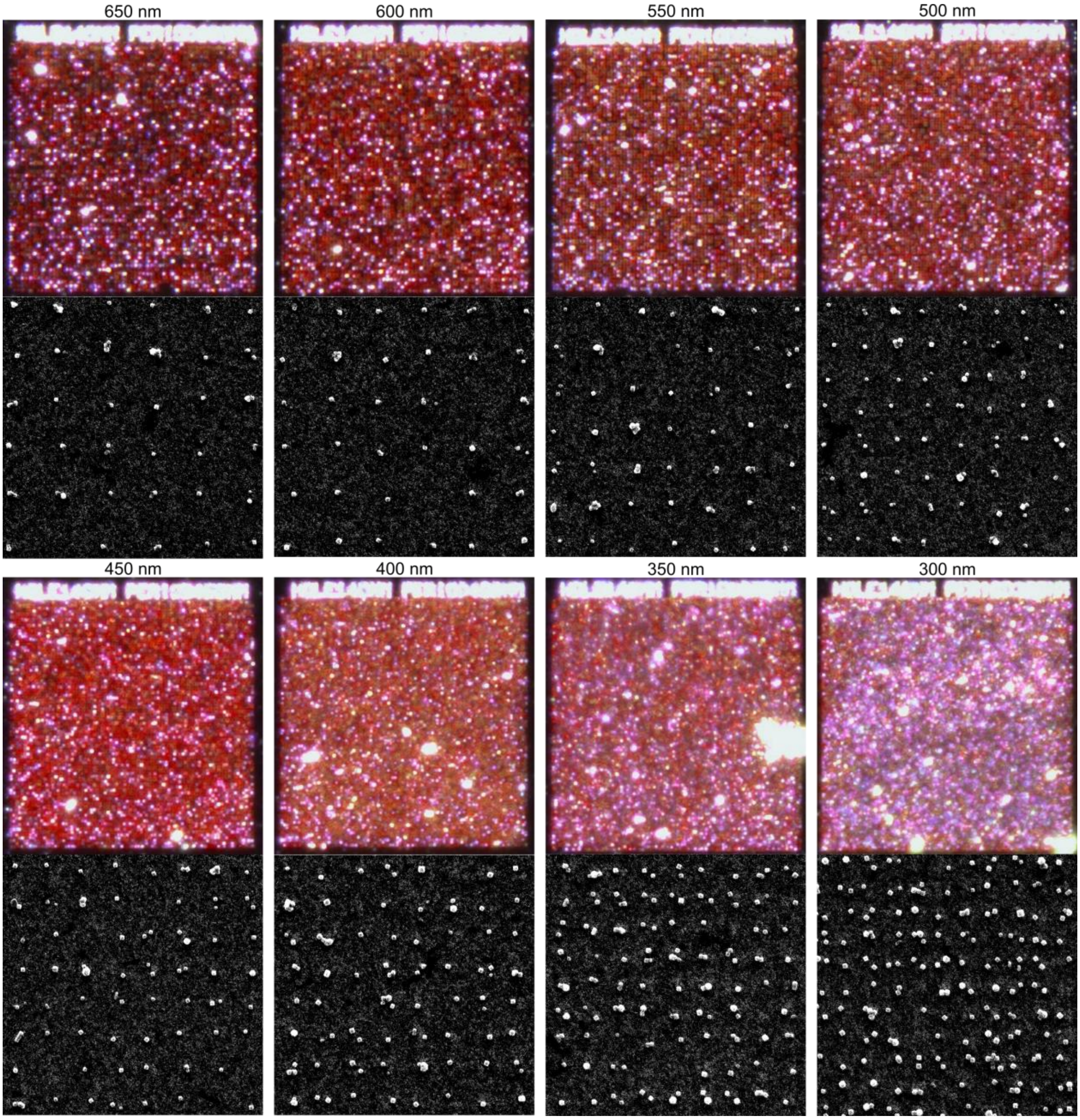

Figure S21. An example of patterned AgNC arrays with different periods. The array period is listed on top of the dark field image. The arrays in DF images are 41.6 μm x 44.2 μm. The sizes of the SEM images are 4 μm × 4 μm.

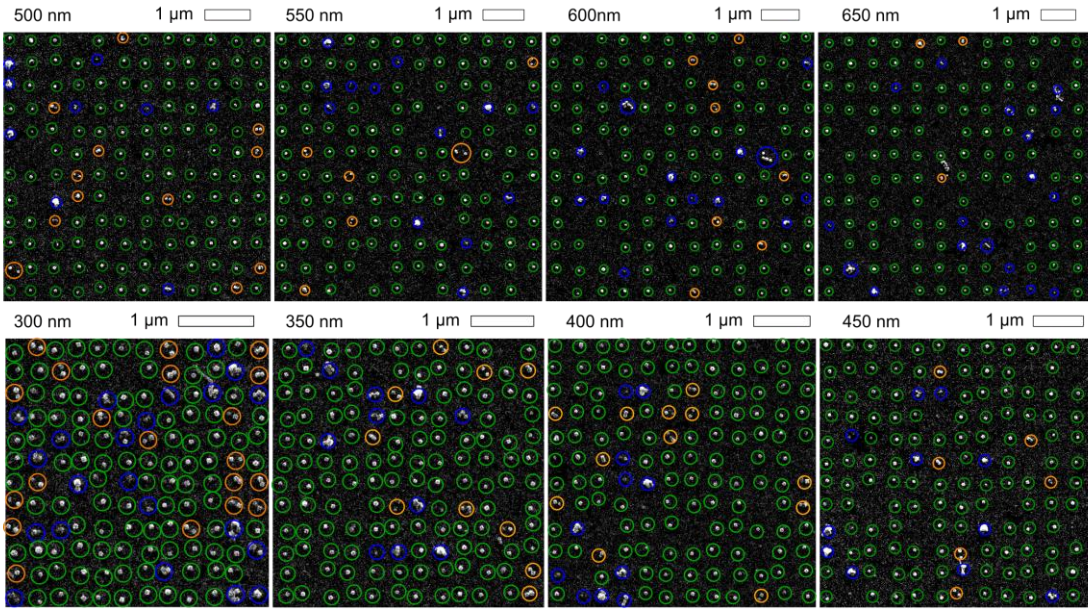

Figure S22. Fill fractions of AgNC arrays with different periods. The array period is listed in the top-left corner. The green, orange, and blue circles correspond to monomers, dimers, and aggregates/undefined particles, respectively. They also match the color bars in Figure S23.

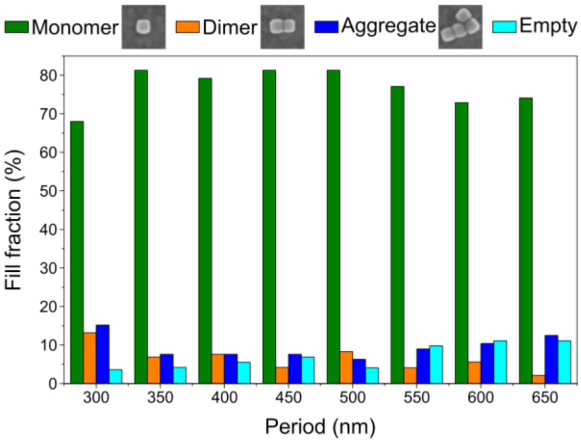

Figure S23. Fill fractions (monomer, dimer, aggregate, empty) vs different array periods. The colors match the circles in Figure S22.

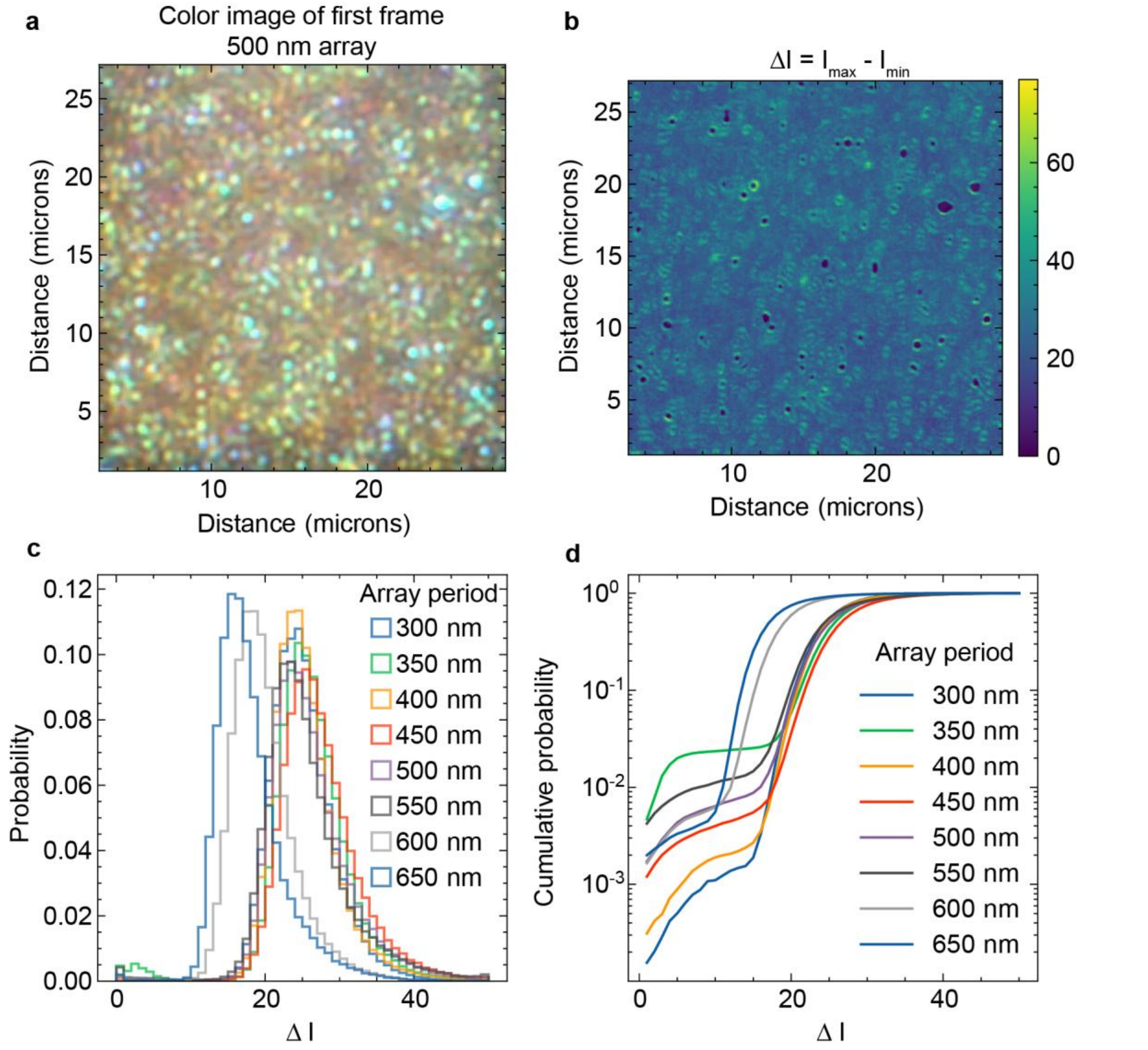

Figure S24. Areal switching uniformity of active eNPoM lattice. (a) First frame from the video of the 500 nm period lattice actuation from supplementary Video S6. (b) For each pixel in the frame, we calculate $\Delta I = I_{max}$-I_{min} over time to see how much the pixel actuates. High values indicate a high degree of actuation, and low values indicate the pixel does not actuate. (c) Histogram of ΔI for each lattice size shown in Video S6. (d) Cumulative probability from the histograms to approximate a cutoff threshold and the percentage of points below the cutoff threshold.

7. AgNC synthesis and characterization

A polyol synthesis procedure was used to prepare the AgNCs. First, the glassware was cleaned for 30 min with an aqua regia solution (HNO_3 + 3 HCl) and then rinsed with Type-1 water (25 °C, 18.2 MΩ cm) and dried with N_2. In a round-bottom flask, 20 mL of ethylene glycol (EG, 99%+ extra pure, Acros Organics) was added. The flask was placed in an oil bath at 150 °C, and the solution was stirred at

300 revolutions per minute (rpm) using a temperature-controlled magnetic stirrer (AREX-6 Digital Pro, VELP® Scientifica Srl). After 40 min, 240 μL of sodium hydrosulfide hydrate ($NaSH{\cdot}xH_2O$, 3 mM in EG, 56.06 g mol^{-1}, Sigma-Aldrich) was added to the solution. One minute later, 2.1 mL of hydrochloric acid (HCl, 3 mM in EG, MW ~ 36.46 g mol^{-1}, Fisher Scientific) was added into the flask and 5 mL of poly (vinylpyrrolidone) (PVP, 20 mg mL^{-1} in EG, MW ~ 55,000 g mol^{-1}, Sigma-Aldrich) immediately after. Next, 1.6 mL of silver trifluoroacetate (CF_3COOAg, 282 mM in EG, Sigma-Aldrich) was added to the solution after two minutes. The flask was sealed with a glass stopper throughout the process. The reaction completed after two hours. Later, the flask containing the AgNC solution was quenched in an ice bath to cool it down. The mixture was then centrifuged using an electric lab centrifuge (Megafuge™ 16R Centrifuge, Thermo Fisher Scientific) at 17,000 relative centrifugal force (rcf) for 12 min at 20 °C. Subsequently, the supernatant was removed, and the pellet was washed with acetone (99.5%, MW ~ 58.08 g mol^{-1}, Acros Organics). The AgNC solution was centrifuged again under the same conditions. However, this time, after removing the supernatant, the pellet was washed with Type-1 water. Thereafter, the AgNC solution was stored in a sealed vial at 4 °C after being redispersed in 7 mL of Type-1 water.

The AgNCs spectrum was measured from 230 to 830 nm with a spectrophotometer (BioSpectrometer®, Eppendorf). For the blank solution, Type-1 water was used. For this measurement, a 10 mm cuvette was utilized. Figure S25 shows the UV absorption of 80× diluted AgNC stock solutions used in the paper. The concentration of AgNC solutions (c) was calculated using the Beer-Lambert Law,

$$\boldsymbol{c = \frac{A \cdot d}{\epsilon \cdot l}}$$

where ϵ is the molar extinction coefficient of AgNC $(4.06\ n\text{M}^{-1}\text{cm}^{-1})$, *A* is the absorbance at the resonant peak, *d* is the dilution factor (*i.e.*, here it is typically 80), and l is the path length in cm (~ 1). The UV-Vis spectra of the synthesized AgNCs are shown in Figure S5. The molar extinction coefficient was determined by measuring the total silver concentration using inductively coupled plasma optical emission spectrometry (ICP-OES, 5900 SVDV, Agilent Inc.) and converting to particle concentration using the known particle dimensions and the FCC crystal structure of silver.

To characterize the morphology of the AgNCs, we utilized a transmission electron microscope (FEI Tecnai 12). A carbon-coated copper TEM grid was drop-cast with 5 µL of the AgNCs solution. The images were captured at 120 kV, and ImageJ Fiji was used to determine the average edge length of the AgNCs. Figure S5 shows TEM images of the used AgNCs, along with their corresponding size distributions. The Gaussian distribution (y) was calculated as follows:

$$\boldsymbol{y = \frac{1}{w\sqrt{\pi/2}} \cdot e^{-2\frac{(x-x_c)^2}{w^2}}}$$

where x is the value of the variable or data being examined, x_c is the mean and w is the standard deviation.

8. Functionalizing gold surfaces with DNA oligos and mercapto-hexanol

Silicon wafers (100 mm, 525 µm thickness, p-doped, 4<100>, Prime grade) were purchased from Siegert Wafers GmbH. Non-patterned gold surfaces were fabricated by evaporating 5 nm titanium and 50 nm gold films using physical vapor deposition (PVD System Ångstrom, 09340, Ångstrom Engineering). We employed standard UV lithography processes to pattern “Aalto logos” on silicon wafers and evaporated the

same 5 nm Ti and 50 nm Au layers. AZ 5214E (Microchemicals GmbH) photoresist was spin-coated to a thickness of 1.5 microns (4000 rpm) on the silicon wafer and baked at 90 °C for 1 min, and the Aalto logo pattern was exposed using UV maskless lithography (Heidelberg Instruments MLA150, 405 nm, 200 mJ cm^{-2} fluence). The exposed pattern was developed using AZ 351B developer for 90 s, followed by a 90 s wash in deionized water. The wafer was dried, and the titanium and gold layers were evaporated on top. Liftoff was carried out by placing the metal-coated wafer in acetone for 2 h at room temperature. The wafer was rinsed with acetone and IPA to peel off the leftover metal from the surface.

After the deposition (and liftoff, if applicable), the wafers were diced into 30 mm × 10 mm chips using a dicing saw (DAD3220, Disco). A plasma cleaner (75 W Tergeo, TG100, PIE Scientific) with a water vapor delivery kit (A122, PIE Scientific) was used to clean and activate the gold surfaces for 2 mins in direct mode at 75 W, using a mixture of air and water vapor. Before coating the Au surface with DNA and 6-Mercapto-1-hexanol (MCH, Sigma-Aldrich), the chips were rinsed with acetone and isopropanol and dried under N_2. 15 µL of 10 mM Tris(2-carboxyethyl)phosphine (TCEP, Sigma-Aldrich), 2.87 mg in 1 mL Type-1 water. Type-1 water (25 °C, 18.2 MΩ cm) and 100 µM substrate strand 9 nt (SH-GTCGAGAGA) were mixed in 5 mL tubes. The DNA-TCEP solution was incubated for 1 hour at room temperature, then 2660 µL of Type-1 water and 296 µL of 10 mM MCH were added to the solution and mixed. Au chips were placed inside the tube and incubated for at least 24 hours.

9. DNA coating of silver nanocubes

We employ the freezing method[3,4], whereby the excluded volume of crystalline ice forces AgNCs and thiolated DNA into high local concentrations for efficient

functionalization and coating of the silver nanocubes with DNA. In contrast, the gold substrate functionalization is achieved by incubation in a DNA-MCH solution to form a mixed self-assembled monolayer (SAM). A solution of DNA-coated AgNCs is then pipetted onto the Au surface, where AgNCs tether *via* hybridization of the complementary DNA linkers. The DNA-functionalized AgNCs and Au surface together form the eNPoM, enabling flexible, robust binding of AgNCs and setting their range of motion. Before use, AgNCs were sonicated for 1 min at room temperature in an ultrasonic washer (M3, FinnSonic Oy) using standard mode. DNA oligos were acquired from Biomers GmhB. Our modified DNA coating protocol is based on previously reported protocols[4], where DNA is mixed with nanoparticles, the mixture is frozen, then thawed after a set time, and purified. The non-patterned samples were fabricated using 452 nm AgNCs, whereas the patterned samples were fabricated with 460 nm AgNCs. The passivating strand is HS-ATCGTACTA. The different anchor strands are HS-GACTAT-$(TC)_x$, where x is 3, 7, and 10 for 12 nt, 20 nt, and 26 nt strands, respectively. To coat the particles, we first mix 8874 µL of Type-1 water, 1050 µL of 0.2% sodium dodecyl sulfate (SDS, ≥ 98.5%, MW ~ 288.38 g mol^{-1}, Sigma-Aldrich), 127.5 µL of AgNC (100 nM), and 400 µL of ssDNA solution in a 15 mL plastic tube. The DNA solution contains 360 µL of Type-1 water, 36 µL of passivating DNA (1 mM), and 4 µL of “anchor” strand (100 µM) so that the ratio between passivating DNA and “anchor” strand is 90:1. The mixture was pipetted into ten 1.5 mL tubes and frozen overnight. After thawing, the tubes were centrifuged with an electric lab centrifuge (Megafuge™ 16R Centrifuge, Thermo Fisher Scientific) at 16,000 relative centrifugal force (rcf) for 11 min. The supernatant was removed, and the tubes were filled to approximately 1 mL with 0.02% SDS. The process was repeated three more times. After the fourth spin, the supernatant was removed, and

the solutions were collected into one tube. The absorption was measured. Figure S25 shows the absorption spectra of AgNC coated with passivating and anchor strands of either 12, 20, or 26 nt. Table S1 shows the corresponding concentrations and yields after coating for each sample. The concentration of the DNA-functionalized AgNCs is estimated using the following equation (S1). It should be noted that, for such calculations, the unnormalized absorbance values were used (*i.e.*, in Table S1). The concentrated DNA-coated AgNCs were stored at 4 °C until use.

Zeta potential measurements were performed using an electrophoretic light scattering (ELS) analyzer (Zetasizer Nano ZS, Malvern Panalytical) with disposable folded capillary cells (DTS1070). Samples of bare (PVP-coated) AgNCs and DNA-functionalized AgNCs (average edge length 56.6 ± 3.7 nm) were diluted 50-fold in ethanol (~ 0.677 nM). Ethanol was used as the dispersant because PVP-coated AgNCs are more stable in ethanol than in water, yielding more reliable measurements. Measurements were conducted at 25 °C with an equilibration time of 120 s. Zeta potential was calculated from the electrophoretic mobility using the Smoluchowski approximation. We note that zeta potential values measured in ethanol ($\varepsilon_{\mathrm{r}} \approx 24.5$) may differ quantitatively from those in aqueous buffer ($\varepsilon_{\mathrm{r}} \approx 78.5$); however, the near-tripling of ζ upon DNA functionalisation (from −6.05 to −16.07 mV) confirms that the DNA dominates the particle's surface charge regardless of the dispersant. The results are shown in Figure S25b.

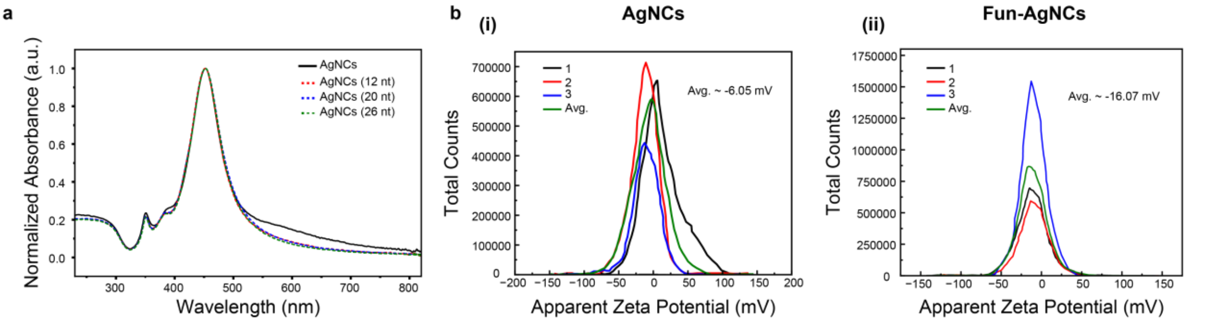

Figure S25. Comparison between AgNCs before and after DNA functionalization. (a) Normalized UV-Vis absorbance of DNA functionalized AgNCs. AgNCs and functionalized AgNCs (Avg. edge length ~ 55 nm) with DNA passivating and "anchor" strands of 12 nt, 20 nt, and 26 nt. (b) Zeta potential for ~ 55 nm AgNCs (452 nm) used in our eNPoM: (i) Zeta potential for unfunctionalized AgNCs. (ii) Zeta potential for DNA-functionalized AgNCs. The 1, 2, and 3 represent multiple measurements, and Avg. indicates the averaged curve.

Table S1. Analysis of the concentration and yield of DNA-functionalized AgNCs with "anchor" lengths of 12, 20, and 26 nt.

	12 nt Linker	20 nt Linker	26 nt Linker
**Concentration (nM)**	28.8	38.1	54.8
**Yield (%)**	26.8	46.5	50.9

Estimation of anchor strand density per AgNC face

To estimate the number of tethering anchor strands in each eNPoM junction, we consider both surfaces separately. On the gold surface, applying the Herne & Tarlov[5] packing density for thiolated ssDNA on flat gold ($5.2 \pm 0.8\times10^{12}$ molecules/cm^2) to the area beneath one AgNC gap-facing facet (55×55 nm^2 = 3.025×10^{-11} cm^2) gives: $(5.2\times10^{12}) \times (3.025\times10^{-11}) \approx 157$ substrate strands beneath one AgNC facet. We note that the gold surface is co-incubated with MCH at ~2000:1 molar ratio (296 μL × 10 mM MCH vs 15 μL × 100 μM DNA), which likely reduces the surface DNA density below this saturation value. Nevertheless, even at a 10-fold lower density, the gold surface would still provide ~16 substrate strands beneath each AgNC footprint, far exceeding the anchor count on the AgNC side. The gold side is therefore not the limiting factor.

To determine the DNA loading on the AgNC surface, we performed a DTT displacement experiment on a batch of DNA-functionalized AgNCs (26 nt anchor) prepared using the same protocol. AgNCs (LSPR 457 nm, average edge length 55.6 ± 5.6 nm, concentration 70.94 nM) were functionalized using the standard freezing-directed protocol described above, with the same DNA volumes (36 µL of 1 mM passivating DNA + 4 µL of 100 µM anchor DNA, 90:1 molar ratio, yield ~ 93.9%). The Fun-AgNCs (66.6 nM) were incubated with 0.5 M DTT in 0.1×PBS (pH 7.4, 1.19 mM phosphates, 13.7 mM NaCl, 0.27 mM KCl) for 12 h at room temperature with shaking (~ 300 rpm) to displace all surface-bound DNA. 0.1×PBS was used instead of the displacement solution containing 4.6 µL of Fun-AgNCs and 38.4 µL of 0.56 M DTT (total 43 µL; final concentrations ~ 7.12 nM AgNCs, ~ 0.5 M DTT). After incubation, the solution was centrifuged at 21,100 rcf for 10 min. 30 µL of supernatant was collected and diluted 10-fold.

The released ssDNA was quantified using a SYBR™ Green II fluorescence assay (S7564, Invitrogen™). The optimal dye-to-sample volume ratio was determined to be 10:1. Eleven calibration standards (0-8,991 nM total ssDNA, anchoring and passivating in 1:90 ratio) were prepared in the same 0.5 M DTT / 0.1×PBS matrix using the same DNA stocks (100 µM anchoring, 1 mM passivating). Fluorescence was measured using a BioTek Synergy H1 plate reader (Agilent) with Gen6 software in a 384-well flat-bottom plate, with excitation at 494/10 nm and emission at 515/10 nm. Measurements were repeated at 0, 5, 10, 15, 20, 25, and 30 min. Values stabilized after approximately 15 min, the plateau average (t = 15–30 min) was 4,346 ± 60 total ssDNA strands per AgNC, corresponding to ~ 48 anchor and ~ 4,298 passivating strands per AgNC (~ 8 anchor and ~ 716 passivating per face).

This sparse anchoring is by design. The passivating strand dilutes the anchor density to provide the particle with mechanical freedom to move under the applied electric field, while maintaining sufficient tethering for stable attachment. The freezing process is stochastic, so individual cube faces will carry more or fewer anchors than the mean.

Electrostatic force analysis

The theoretical framework for electrically actuated surface-tethered DNA was established by Rant *et al.*[6] who demonstrated dynamic switching of DNA orientation on gold electrodes, and formalized quantitatively by Langer *et al.*[7] who showed that the electrostatic driving force acts primarily on the DNA backbone within the high-field region of the electrochemical double layer, while a bulky label at the DNA end contributes mainly to hydrodynamic drag. We apply this reasoning to our eNPoM system by comparing the charges on the DNA and on the bare particle.

DNA charge

Each nucleotide in ssDNA carries one elementary charge (1 *e*) from its phosphodiester group. From the DTT displacement measurement above, the gap-facing facet (~ 55 × 55 nm^2) carries ~ 724 total strands (~ 8 anchor at 26 nt, ~ 716 passivating at 9 nt). The total bare DNA charge on one facet is (716 × 9) + (8 × 26) = ~ 6,652 *e*.

Particle charge from Grahame equation

As a quantitative cross-check, we estimate the charge on the bare AgNC gap-facing facet using the linearized Grahame equation[8]:

$$\sigma = \varepsilon_0 \cdot \varepsilon_r \cdot \kappa \cdot \psi_0$$

where σ is the surface charge density (C/m²), ε_0 is the vacuum permittivity (8.854×10^{-12} F/m), ε_r is the relative permittivity of water (~ 80), κ is the inverse Debye screening length, and ψ_0 is the surface potential, approximated here by the measured zeta potential (ζ = 6.05 mV). This linearised form is valid when $e\psi_0/2kT \ll 1$ (i.e., when the surface potential is much smaller than $2kT/e$ ≈ 51 mV at 25 °C). (where k is the Boltzmann constant, *T* is the absolute temperature, and e is the elementary charge). Our ζ = 6.05 mV satisfies this condition. The Debye length is $\kappa^{-1}\ (nm) \approx 0.304/\sqrt{c}$ for a 1:1 electrolyte at 25 °C, where *c* is the molar concentration. We note that ζ is measured at the hydrodynamic slipping plane rather than at the particle surface itself, so this estimate is conservative (it underestimates the true surface charge). The charge on one facet is:

$$Q = \sigma \cdot A / e$$

where *A* = $(55\text{ nm})^2 = 3.025\times10^{-15}$ m². At ~ 1 mM (low added NaCl), κ^{-1} = 9.6 nm and $\kappa = 1.04\times10^{8}$ m^{-1}. Therefore,

$$\sigma = (8.854 \times 10^{-12}) \cdot (80) \cdot (1.04 \times 10^{8}) \cdot (6.05 \times 10^{-3}) = 4.46 \times 10^{-4}\ C/m^2$$

$$Q = \frac{(4.46 \times 10^{-4}) \cdot (3.025 \times 10^{-15})}{1.602 \times 10^{-19}} \approx 8\,e$$

At 100 mM NaCl, κ^{-1} = 0.96 nm and $\kappa = 1.04\times10^{9}$ m^{-1}. Thus, $\sigma = 4.46\times10^{-3}$ C/m² and $Q \approx 84\ e$.

Comparison

The DNA bare charge per facet (~ 6,652 *e*) exceeds the estimated intrinsic particle charge per facet (~ 8 - 84 *e*, depending on ionic strength) by approximately 79× to 832×. Even acknowledging that the Grahame estimate underestimates the true particle charge (since $\zeta < \psi_o$), the DNA charge dominates by at least two orders of

magnitude across all operating conditions. The electrostatic driving force, therefore, acts overwhelmingly on the DNA backbone rather than on the particle itself.

Manning counterion condensation correction

Manning counterion condensation reduces the effective charge on a polyelectrolyte when the dimensionless Manning parameter ($\xi = l_B/b$) exceeds 1, where $l_B = e^2/(\varepsilon kT) = 7.135$ Å is the Bjerrum length in water at 25 °C and b is the axial distance between charged groups.[9] For ssDNA, b equals the length per nucleotide. Using b = 6.3 Å,[10] $\xi = 7.135/6.3 = 1.13$ and the neutralized fraction is $(1 - 1/\xi) = 12\%$, leaving 88% effective charge (~ 5,854 *e* per facet). Even after condensation, the DNA charge exceeds the particle charge (~ 8-84 *e*) by at least 70×, confirming that the DNA backbone is the dominant site of electrostatic force.

Anchoring stability under applied bias

The applied field could, in principle, weaken the thiol-Au bonds or DNA hybridization that hold the system together. Three observations indicate that anchoring remains intact under our operating conditions.

i. The switching is fully reversible over 100s of cycles at < 0.5 V (Figure S17).
ii. The spectral modulation increases systematically with anchor strand length (12 → 20 → 26 nt, Figures 2, S1, S2), consistent with longer tethers providing a greater range of motion rather than with bond disruption.
iii. The modulation is ionic-strength-dependent (Figure 2d), as expected for a double-layer-mediated mechanism rather than an electrochemical degradation process.

Operating below 0.5 V avoids electrochemical desorption of thiolated DNA from gold, which has been shown to begin weakly around -0.4 V and reach a sharp onset at -

0.65 V vs Ag/AgCl.[11,12] We note that the desorption thresholds above were measured against an Ag/AgCl reference electrode, whereas our system applies a voltage between the gold film and an ITO counter electrode without a formal reference. The actual potential at the gold-electrolyte interface, therefore, depends on the ITO offset potential and the ohmic drop across the electrolyte, making a direct quantitative comparison approximate. Nevertheless, the three experimental observations listed above, in particular the fully reversible switching over hundreds of cycles (Figure S17), provide direct evidence that anchoring remains intact under our operating conditions regardless of the precise electrochemical potential at the gold surface.

Calculation of eNPoM switching energy

We can assume that during switching, the DNA layer transitions from an extended to a compact state (or *vice versa*), changing the effective linear charge density in the NC-substrate gap. We can begin with the Guoy Chapman potential in monovalent electrolyte $\phi(z,c,T,\epsilon) = 2\frac{k_B T}{e}\log\left(\frac{1+\gamma e^{-\frac{z}{\lambda_D}}}{1-\gamma e^{-\frac{z}{\lambda_D}}}\right)$ where $\lambda_D(\epsilon,T,c) = \sqrt{\frac{\epsilon\epsilon_0 RT}{2*1e3*F^2 c}}$ is the Debye length, ϵ is the dielectric constant of the electrolyte, T is the temperature in Kelvin, c is the concentration of electrolyte in mol/L, $\gamma = \tanh\left(\frac{e\phi}{4k_B T}\right)$, ϕ is the potential applied to the electrode, e is the electron charge, R is the molar gas constant, and k_B is the Boltzmann constant. We then calculate the electrostatic potential energy U of the linear charge density λ_{DNA} in the electrostatic double layer $U = \int_0^L \lambda_{DNA}(z)\,\phi(z)dz$ where L is the approximate size of the gap. The electrode potential is multiplied by a dielectric screening parameter ~0.1 because approximately 90% of the potential drops already across the MCH spacer layer[7]. Using 10 mM monovalent salt, ε = 80, DNA length 28 base pairs, 300 strands per

cube face, $\pm 300\, mV \cdot (0.1)$ with corresponding 5 nm and 15 nm NC-substrate gaps, $0.24 \cdot 2 \cdot e$ charge per base pair (accounting for counter-ion condensation), and assume the charge density is linear across the gap, electrostatic potential energy difference between positive and negative states is less than 0.02 fJ. This estimate may vary due to the exponential decay of the potential in the gap region, the approximation of substrate-gap distances, electrode potential, and amount of gap DNA.

10. Attachment of AgNC on the DNA-MCH-Au surface

After 24 hours of incubation, the DNA-MCH-Au surface was rinsed with 3 × 100 µL of Type-1 water (25 °C, 18.2 MΩ cm). Excess liquid was blotted away using a disposable lab wipe. 100 µL of AgNC solution was then added to the surface and incubated for 1.5 hours. During deposition, the AgNCs and the AuNCs concentrations were fixed at 6.8 and 0.4 nM, respectively, and the buffer was set to 0.5×TE and 100 mM NaCl. This means that we typically fixed 10 µL of 5×TE/1 M NaCl buffer with appropriate amounts of AgNCs or AuNCs solutions and Type-1 water to achieve the desired deposition solution. After 1.5 hours of deposition, the gold film typically changed color to light red, and we rinsed the samples with 3 × 100 µL of 0.5×TE and 100 mM NaCl buffer. The samples were stored in the same buffer in the fridge.

The surface of eNPoM was characterized using a scanning electron microscope (SEM, Sigma VP, Zeiss) with an In-Lens detector under high vacuum (< 10^{-5} Pa) and 5 – 7 kV acceleration voltage. Images were analyzed using ImageJ Fiji and MATLAB. The fill fraction (%*FF*) was calculated using the following formula:

$$\%FF = \frac{Area\ of\ particles}{Total\ area} \times 100\%.$$

The %*FF* for 12, 20, and 26 nt "anchor" strand samples were 8.9, 22.6, and 10.9 %, respectively. The average distances between cubes were 276.9 ± 75.9 nm, 302.6 ± 72.7 nm, and 155.4 ± 42.9 nm for 12, 20, and 26 nt "anchor strand" samples, respectively. Figure S26 shows a typical AgNC-covered Au surface with an "Aalto-logo". The average *FF* and distances between cubes for the shown sample in areas (i) and (ii) were 18.1 ± 1.4 %, 199.6 ± 38.8 nm, and 17.2 ± 1.2 %, 197 ± 37.1 nm, respectively. For the AuNCs on Au film samples, the *FF* was calculated as 11 ± 1.8 %, and the average distances between cubes were around 373.5 ± 116.3 nm. Figure S27 shows a representative SEM image of the surface of samples with AuNCs on Au surfaces.

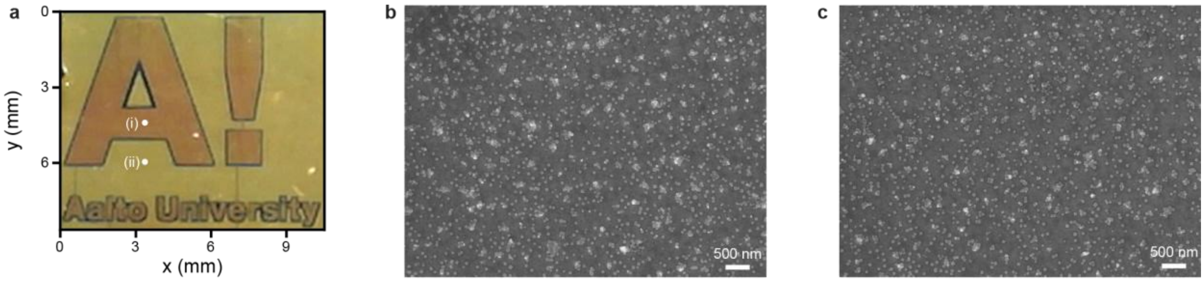

Figure S26. DNA functionalized AgNCs distribution on the surface of an "Aalto logo" sample. (a) A photograph showing the surface of the sample. (b) A representative SEM image of the surface at region (i) in a. (c) A representative SEM image of the surface at region (ii) in a. The average size of the particles is ~ 55.4 ± 8.9 nm. Aalto logo patterned eNPoM. Credit: Aalto University. Logo used with permission.

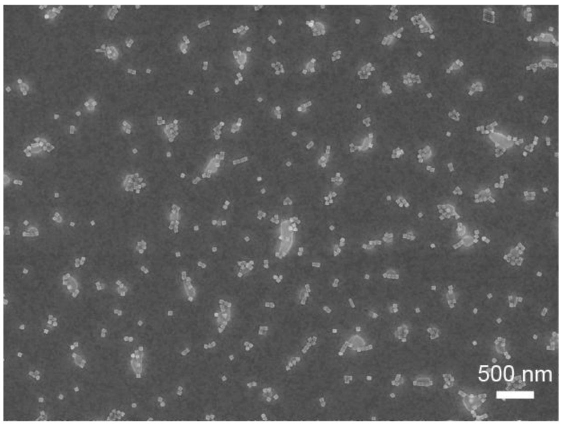

Figure S27. A representative SEM image of DNA functionalized AuNCs distribution on an Au surface. The average edge length of the cubes is ~ 54.5 ± 3.2 nm.

11. eNPoM DC voltage bias reflectance measurement

The samples were loaded into a liquid cell containing a plexiglass cover and base with a polydimethylsiloxane (PDMS) spacer, as shown in Figure S28. The sample was first fixed to the base with copper clamps, the PDMS spacer was placed on the base, and the cell was sealed by placing the cover with an indium tin oxide-coated glass (ITO Glass Substrates, Ossila BV) on top. The copper clamps were used as electric contacts to bias the gold substrate. Copper tape (E17385, thickness ~ 66 μm, 3M™) and conductive glue (8331D-14G, MG Chemicals) were used to make contact between the ITO and the screws holding the cell.

The liquid cell was filled with buffer, sealed, and placed in a spectrophotometer (V-770, Jasco) equipped with an absolute reflectance unit (ARSN-917, Jasco). A normal incidence angle of 6° was used, and a white plate standard of Spectralon® (6708-H006A, Jasco) was used for the baseline spectrum. During measurement, the bandwidth was 5 nm, the scan speed was 2000 nm min^{-1}, and the data interval was 2 nm. We measured the non-biased curve first and connected the power source (AFG-2112, GW Instek) between the gold and ITO. A constant DC bias voltage was applied, and the reflectance was recorded. During reflectance measurements, we cycled between negative and positive voltages to confirm that the samples were active and reversible. After a few cycling steps were recorded, the buffer was

exchanged. The order of measured buffers was from 100 mM to 0 mM NaCl. Each spectrum took roughly 90 seconds to measure.

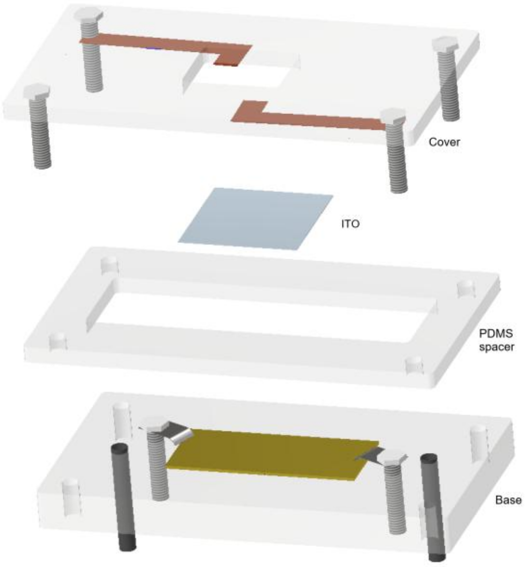

Figure S28. Schematic view of the liquid cell. The top cover has an opening for attaching indium tin oxide (ITO) glass, and copper tape, together with conductive glue, is used to create electric contacts to ITO. The base includes two copper clamps to hold the gold- and titanium-coated silicon chip and to make electric contacts. The PDMS spacer is sandwiched between the cover and base to form the liquid cell.

The conductivity of the buffers was measured using a dual-purpose conductivity/pH/T meter equipped (MPC227, Mettler Toledo) with a conductivity probe (INLAB 731, Mettler Toledo). The conductivities of 0.5×TE, 0.5×TE/10 mM NaCl, and 0.5×TE/100 mM NaCl were 0.51, 1.4, and 11.0 mS cm^{-1}, respectively.

12. eNPoM AC voltage actuation measurements

The high-speed measurements using the liquid cell in Figure S28 were carried out with a high-speed CCD camera (Phantom v1612, Vision Research Inc.) mounted on an optical table. Since the camera is monochromatic, a 560 nm LED (Thorlabs M565L3 with LEDD1B driver) was chosen, since the highest reflectance modulation is achieved at that wavelength across all samples. The liquid cell was positioned in front of the camera, with the LED illuminating the sample at an angle where the signal was maximized at the camera. The voltage source (4047B, B&K Precision

Corporation or Analog Discovery 3, Digilent) was coupled to the liquid holder. The reflected light from the sample was recorded as a function of AC driving frequency (1 Hz to 1 kHz) and buffer conditions (0.5×TE with 0, 10, or 100 mM NaCl). The acquisition parameters are listed in Table S2.

Table S2. High-speed measurement parameters.

F_d (Hz)	Sample rate (fps)	Exposure time (μs)
1	300	300
10	1000	300
100	5000	199
1000	20000	49.4

13. Patterning eNPoM substrates using PMMA hole array masks

Initially, PMMA 950 A2 is spun on top of the gold film using 1600 rpm and 60 s. The sample is baked for 90 s at 180 °C and placed inside the JSM-7100F scanning electron microscope. We use NPGS patterning software to expose and pattern the surface. During patterning, we use an acceleration voltage of 30 kV and a probe current of 1. A typical current is around 35 pA current, measured using a Faraday cup just before the writing. After patterning, the sample is developed in 3:1 = isopropanol (IPA): methyl isobutyl ketone (MIBK) for 60 s, rinsed using IPA, and N_2 dried.

The patterned gold surface is MCH-DNA functionalized, and AgNCs are drop-cast similarly to Figure 1. However, since we use sparser DNA coating on the nanoparticles and metal films than reported in the literature[1,13], each process step was optimized to achieve high patterning yields while retaining reversible actuation. Ideally, when a single particle binds inside a hole in the PMMA mask, it blocks the binding of another particle due to steric hindrance and Coulombic repulsion. For different AgNC patches with varying edge lengths and monodispersity, single particle occupancy is

strongly dependent on the hole size. We tested different hole sizes and found that the most optimal hole size for DNA coated 55 nm AgNC is around 140 nm (see Figures S18 and S19). Larger hole sizes resulted in a higher dimer occupancy.

Samples are plasma-treated for 10 s at 75 W and incubated for 24 h in the same DNA and MCH solution. Afterwards, samples are dipped twice in Type-1 water solutions in a 2 mL tube, excess liquid is blotted away from the backside of the chip, and AgNC solution is drop-cast for 1 h and 20 min. The concentration of AgNC is 60 nM during drop-casting, and the buffer is 0.1 M NaCl, 0.1% SDS, and 2 mM $MgCl_2$. After drop-casting, the surface is rinsed with 5 x 5 mL Type-1 water, dipped in a solution containing 80 % IPA and 20 % acetone, and placed in a 2 mL tube containing 60 % IPA and 40 % acetone. Samples are incubated for 30 min at 40 °C, then washed once by dipping in 0.5×TE and 0.1 M NaCl buffer and stored in the same buffer. To demonstrate electric actuation, we insert samples into the self-made liquid cell, place the cell under an Olympus BX53 upright microscope with an Olympus MXPLANFL N ×50 dark field objective (NA = 0.8), and apply AC square voltage with 150 mV peak voltage between ITO and the gold film. The videos are recorded with a Pixelink M7C-CYL color camera and post-processed in Davinci Resolve (Blackmagic design, version 18.6). During post-processing, we only adjust the contrast and brightness to highlight the actuation. The videos are white balanced using a white reference. After recording the actuation, samples are rinsed once with 300 µL Type-1 water and dried under N_2.

It should also be noted that samples contain larger aggregates (bright spots in the dark field images) that originate from the AgNC solutions. We sonicate the solution before drop-casting to break these aggregates, but prolonged sonication also removes the DNA from AgNC. Using inherently more stable particles, such as

AuNC, could also improve the overall quality of the array, but our results demonstrate the method at a proof-of-concept level.

References

(1) Lin, Q.-Y.; Mason, J. A.; Li, Z.; Zhou, W.; O'Brien, M. N.; Brown, K. A.; Jones, M. R.; Butun, S.; Lee, B.; Dravid, V. P.; Aydin, K.; Mirkin, C. A. Building Superlattices from Individual Nanoparticles via Template-Confined DNA-Mediated Assembly. *Science* **2018**, *359*, 669–672. https://doi.org/10.1126/science.aaq0591.

(2) Elhadj, S.; Singh, G.; Saraf, R. F. Optical Properties of an Immobilized DNA Monolayer from 255 to 700 Nm. *Langmuir* **2004**, *20*, 5539–5543. https://doi.org/10.1021/la049653+.

(3) Liu, B.; Liu, J. Freezing Directed Construction of Bio/Nano Interfaces: Reagentless Conjugation, Denser Spherical Nucleic Acids, and Better Nanoflares. *J. Am. Chem. Soc.* **2017**, *139*, 9471–9474. https://doi.org/10.1021/jacs.7b04885.

(4) Tapio, K.; Mostafa, A.; Kanehira, Y.; Suma, A.; Dutta, A.; Bald, I. A Versatile DNA Origami-Based Plasmonic Nanoantenna for Label-Free Single-Molecule Surface-Enhanced Raman Spectroscopy. *ACS Nano* **2021**, *15*, 7065–7077. https://doi.org/10.1021/acsnano.1c00188.

(5) Herne, T. M.; Tarlov, M. J. Characterization of DNA Probes Immobilized on Gold Surfaces. *J. Am. Chem. Soc.* **1997**, *119*, 8916–8920. https://doi.org/10.1021/ja9719586.

(6) Rant, U.; Arinaga, K.; Fujita, S.; Yokoyama, N.; Abstreiter, G.; Tornow, M. Electrical Manipulation of Oligonucleotides Grafted to Charged Surfaces. *Org. Biomol. Chem.* **2006**, *4*, 3448–3455. https://doi.org/10.1039/B605712H.

(7) Langer, A.; Kaiser, W.; Svejda, M.; Schwertler, P.; Rant, U. Molecular Dynamics of DNA–Protein Conjugates on Electrified Surfaces: Solutions to the Drift-Diffusion Equation. *J. Phys. Chem. B* **2014**, *118*, 597–607. https://doi.org/10.1021/jp410640z.

(8) Israelachvili, J. N. *Intermolecular and Surface Forces*, 3rd ed.; Academic Press, 2011.

(9) Manning, G. S. Limiting Laws and Counterion Condensation in Polyelectrolyte Solutions I. Colligative Properties. *J. Chem. Phys.* **1969**, *51*, 924–933. https://doi.org/10.1063/1.1672157.

(10) Murphy, M. C.; Rasnik, I.; Cheng, W.; Lohman, T. M.; Ha, T. Probing Single-Stranded DNA Conformational Flexibility Using Fluorescence Spectroscopy. *Biophys. J.* **2004**, *86*, 2531–2537. https://doi.org/10.1016/S0006-3495(04)74308-8.

(11) Takeishi, S.; Rant, U.; Fujiwara, T.; Buchholz, K.; Usuki, T.; Arinaga, K.; Takemoto, K.; Yamaguchi, Y.; Tornow, M.; Fujita, S.; Abstreiter, G.; Yokoyama, N. Observation of Electrostatically Released DNA from Gold Electrodes with Controlled Threshold Voltages. *J. Chem. Phys.* **2004**, *120*, 5501–5504. https://doi.org/10.1063/1.1643729.

(12) Arinaga, K.; Rant, U.; Knežević, J.; Pringsheim, E.; Tornow, M.; Fujita, S.; Abstreiter, G.; Yokoyama, N. Controlling the Surface Density of DNA on Gold by Electrically Induced Desorption. *Biosensors and Bioelectronics* **2007**, *23*, 326–331. https://doi.org/10.1016/j.bios.2007.04.012.

(13) Zhang, H.; Cadusch, J.; Kinnear, C.; James, T.; Roberts, A.; Mulvaney, P. Direct Assembly of Large Area Nanoparticle Arrays. *ACS Nano* **2018**, *12*, 7529–7537. https://doi.org/10.1021/acsnano.8b02932.
